# Deserts, Rivers, Pools, and Billabongs: Water Features of the Nitrogenase Proteins, and their Functions

**DOI:** 10.1002/cbic.202500541

**Published:** 2025-09-29

**Authors:** Ian Dance

**Affiliations:** ^1^ School of Chemistry UNSW Sydney Kensington 2065 Australia

**Keywords:** enzyme catalysis, mechanism, nitrogenase, protein structures, water chemistry

## Abstract

This review examines the occurrence and function of water inside the protein that contains the catalytic site of the enzyme nitrogenase. The requirement of 8 protons and 8 electrons to convert N_2_ to NH_3_ and the opposing acid—base character of reactant and product are unique in enzymology. The active site is an unprecedented iron sulfide cluster containing one heterometal, in Mo, V, and Fe isozymes. A key component supporting the complex chemical mechanism is water, which transports multiple exogenous protons, sequentially, and assists the egress of hydrophilic ammonia. Using high‐resolution crystal structures of the nitrogenase isozymes and cryoEM data, I describe and classify all intraprotein water components. A singular property is the occurrence of extensive anhydrous domains that surround the reaction zone of the cofactor. This focuses attention on the proton supply chain, a river, along which protons are transferred by a Grotthuss mechanism from protein surface to cofactor. Another river, in an opposite direction, runs along the pathway for departing NH_3_. I describe mechanisms for translocation of protons and of NH_3_ and their use of water and homocitrate. Other water features buried in the proteins include a mechanistically significant single water molecule and featureless water pools.

## Graphical abstract

In the protein surrounding the active site of nitrogenase there is a large anhydrous desert, traversed by two watercourses necessary for the enzyme to convert N_2_ to NH_3_. Various pools exist, but their water seems to be stagnant.

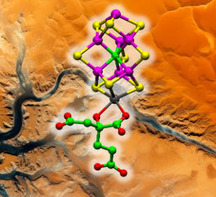

## 1 Introduction

Nitrogenase is the enzyme that converts N_2_ to NH_3_, an inherently difficult chemical transformation. Discovered in 1888, biological nitrogen fixation was first investigated in many laboratories with affiliations reflecting its agricultural importance.^[^ ^1^ ^]^ Following preparation of purified samples of the bacterial enzyme in the early 1960s, a large collection of phenomenological data was accumulated, accompanied by hypotheses about the mechanism of N_2_ activation and conversion.^[^ ^2^ ^]^ Research was stimulated by the revelation of the enzyme structure, via crystal diffraction, in the 1990s. The active site of the enzyme is a unique metal sulfide cluster, now known to be centered with a C atom.^[^ ^3^, ^4^, ^5^, ^6^, ^7^ ^–^ ^8^ ^]^ More recent research on nitrogenase is driven by, amongst other factors, the occurrence of many steps in the complete mechanism, the unprecedented chemistry of the catalytically active site, and the fact that the enzyme achieves under ambient conditions a difficult chemical transformation that the best industrial or abiological processes still cannot achieve. Theory and computational simulation have led to postulated mechanisms,^[^ ^9^ ^]^ but experimental confirmation is frustrated by difficulties in trapping under physiological conditions the many intermediates in the mechanistic cycle.^[^ ^10^ ^,^ ^11^ ^]^

In this review, I study a neglected aspect of the enzyme, which is the occurrence of water molecules within the nitrogenase protein. There are three isozymes, Mo‐nitrogenase, V‐nitrogenase, and Fe‐nitrogenase, using one of these three metals in the catalytic cofactor. Each isozyme is comprised of two component proteins. The smaller protein which contains only FeS clusters is named the Fe‐protein or component 2. This component (also labeled according to function, as dinitrogenase reductase) controls the ATP hydrolysis and coupled reduction of its FeS cluster. The larger protein, component 1, contains the catalytically active cofactor named FeMo‐co, FeV‐co, or FeFe‐co. Component 1 also contains another FeS cluster, the P‐cluster, controlling electron transfer from the Fe‐protein to the catalytic cofactor. The interesting water features exist in the component 1 protein of each isozyme, labeled in the following as the MoFe, VFe, or FeFe protein.

Consider the stoichiometry of the nitrogenase reaction effected by Mo‐nitrogenase under optimal conditions (Equation 1). The requirement of a large number, 8, of protons and electrons confers unique status in enzymology. Less efficient V‐nitrogenase uses even more, 12 electrons and protons, to produce 2NH_3_ and 3H_2_ from N_2_.^[^ ^12^ ^]^ Mo‐nitrogenase has 17 species as reactants and 3 products and so appears to need routes and traffic control for 20 components.

(1) $$N_{2}\;+\;8e^{-}\;+\;8H^{+}\;\to \;2{\mathrm{NH}}_{3}\;+\;H_{2}$$

This daunting realization is ameliorated by the insight of Colin Wraight.^[^ ^13^ ^]^ Wraight clearly describes how electron transfer is controlled by potential differences, not structural aspects of pathways. This principle operates in nitrogenase through a reduction cycle in which energy‐supplying ATP hydrolysis occurs in the Fe protein, which docks with component 1 protein, inducing geometrical changes at the P‐cluster, modifying its potential, and affecting the timing of electron transfer to the catalytic cofactor. However, attempts to define a pathway for this transfer of an electron from the P‐cluster, through intervening protein, to the catalytic cofactor, appear to have limited value.

Proton transfer mechanisms are distinctly different. Regarding proton supply in general, Wraight states “In contrast to electron transfer, proton transfer is *exquisitely dependent on structure*.”^[^ ^13^ ^]^ While the prospects for understanding pathways for passage of eight electrons through intervening protein to the catalytic cofactor are limited, detailed descriptions of mechanism for the supply of eight protons to the cofactor are accessible and should be evident in the protein structure.

The nitrogenase reaction has another distinctive characteristic. While reactant N_2_ and product H_2_ are aprotic, the reactant H^+^ and product NH_3_ are distinctly protic and on opposite sides of the acid–base conflict. The enzyme uses an acidic reactant to generate a basic product, which adds another requirement for the traffic control.

Enter water. A chain of water molecules is well established as a structural component for translocation of protons in biochemical and other systems^[^ ^14^, ^15^, ^16^, ^17^, ^18^, ^19^ ^–^ ^20^ ^]^ and is the fundamental agent of acid–base chemistry.

The considerations above are the impetus and platform for this analysis of the water structure of nitrogenase proteins. Previous analyses^[^ ^21^, ^22^ ^–^ ^23^ ^]^ are extended, and comparisons with the V and Fe nitrogenase isozymes^[^ ^24^ ^]^ are included. The protein crystal structures examined in this analysis are detailed in **Table** **1**. These crystal structures were examined because they appear to contain a fully resolved set of water molecules, as judged by the number of independent resolved water molecules per independent unit, i.e., per cofactor catalytic site. This number is about 1300, with variations (±100) probably due to differences in resolving water molecules on the protein surfaces. The single structure of the FeFe protein (8BOQ) reports considerable fewer waters. For the other structures in Table 1, it is assumed that all intraprotein waters have been resolved crystallographically and are included in the PDB files. The exogenous inclusions, derived from the crystallization medium, are all hydrophilic and significant because they reveal internal regions of protein accessible from the surface and therefore regions that are available to surface protons.

**Table 1:** Well‐resolved crystal structures of the nitrogenase component 1 protein in Mo‐, V‐, and Fe‐nitrogenase, analyzed in this report.

PDB speciesa)	Crystallization conditions	Resolution	No. of independent units	No. of water molecules per independent unit	Exogenous inclusions (per independent unit)	References
**Mo nitrogenase**						
3U7Q *Av*1	pH 8.0, 292K	1.0Å	2	1301	Imidazolium ions (4) Mg^2^^+^ (1) Ca^2^^+^ (1)	[5]
4WES *Cp*1	295K	1.08Å	2	1242	2‐methyl‐2,4‐pentanediol (0.5)	[64]
1QH8 *Kp*1	mixed oxidation state	1.60Å	2	1404	1,2‐ethanediol (8.5) [Mg(H_2_O)_6_]^2+^ (1) [Mg(H_2_O)_5_]^2+^ (1.5) [Mg(H_2_O)_2_]^2+^ (1) Cl‐ (1)	[30]
1QGU *Kp*1	dithionite‐reduced state	1.60Å	2	1368	1,2‐ethanediol (8) [Mg(H_2_O)_6_]^2+^ (2) [Mg(H_2_O)_5_]^2+^ (0.5) [Mg(H_2_O)2]^2+^ (1) Cl‐ (1)	[30]
1QH1 *Kp*1	oxidized	1.60Å	2	1295	1,2‐ethanediol (6.5) [Mg(H_2_O)_6_]^2+^ (2) [Mg(H_2_O)_5_]^2+^ (0.5) [Mg(H_2_O)_2_]^2+^ (1) Cl‐ (1)	[30]
**V nitrogenase**						
5N6Y *Av*1	pH 7.5, 293K	1.35	2	1124	Mg^2^^+^ ion (2)	[31]
6FEA *Av*1	pH 7.5, 293K “turnover state,” contains displaced HS‐	1.20	2	1326	Zn^2^^+^ (1) Mg^2^^+^ (2)	[32]
7ADR *Av*1	turnover under CO, “CO bridged state”	1.00	2	1275	Tris buffer (1.5) Mg^2^^+^ (2) 1,2‐ethanediol (4.5)	[65]
7ADY *Av*1	CO state turned over under Ar → “CO removed state”	1.05	2	1278	Tris buffer (1.5) Mg^2^^+^ (2) 1,2‐ethanediol (4.5)	[65]
**Fe nitrogenase**						
8BOQ *Av*1	Cocrystallization of resting and turnover states	1.55	2	714	Mg^2^^+^ (1)	[33]

a) Species *Azotobacter vinelandii* (*Av*), *Clostridium pasteurianum* (*Cp*), and *Klebsiella pneumoniae* (*Kp*).

## 2 Objectives and Definitions

The objectives of this review are (1) to examine details of the various occurrences of water and water structures within the component 1 nitrogenase proteins, (2) to identify water features that are conserved and therefore likely to be mechanistically significant, (3) to compare the water structures of the isozymes and of different species, (4) to interpret this information in order to understand the mechanisms of proton supply and proton usage in the nitrogenase mechanism, and (5) to further understand the pathway for egress of product NH_3_. This augments previous reports describing the pathway^[^ ^21^ ^]^ and mechanism^[^ ^23^ ^]^ for proton supply to the active site of Mo‐nitrogenase and the pathway and mechanism for NH_3_ egress in Mo‐nitrogenase.^[^ ^22^ ^]^ This review covers ancillary and supporting aspects of the complete chemical mechanism, proposed separately,^[^ ^25^, ^26^, ^27^ ^–^ ^28^ ^]^ for catalysis of N_2_ reduction at FeMo‐co.

The metaphors used in describing and differentiating the water features in these proteins need elaboration. Deserts are regions devoid of water molecules. Rivers are chains of water molecules, usually unbranched. Pools are aggregates of water molecules, with limited connections to other features. Billabongs? This is a word from Australian aboriginal language, meaning dead water or dead river. It often describes a stagnant backwater. So, I use it to describe, in proteins, isolated collections of water molecules that are nonfunctional.

Descriptions of mechanism often refer to proton transfers. Proton terminology refers to an H atom, polarized slightly positive by its bond to a more electronegative atom. Protons and H^‐^ ions (2 electrons) cannot exist as such in condensed media, including protein and water, or on atoms of the metal sulfide cluster at the catalytic site.^[^ ^29^ ^]^ It is necessary to reiterate this reality because some descriptions of mechanism have assumed otherwise, incorrectly.

## 3 Navigation Around the Active Site

**Figure 1:**
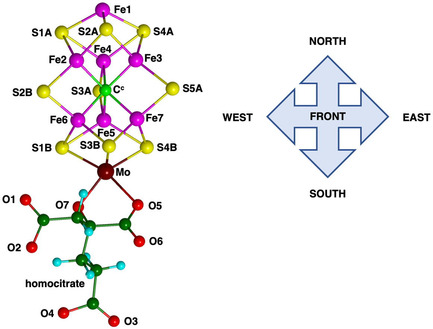
Structure of FeMo‐co and the navigational compass. Additional ligation by cysteine at Fe1 and histidine at Mo is not shown.

Presentation of structure and structural features is primarily pictorial, and the protein structures are large. A spatial orientation framework is needed. Here, pictorial presentations of water in the complete protein extending outwards from the cofactor are based on a standardized view direction normal to the Fe2‐Fe3‐Fe6‐Fe7 face of the cofactor cluster and from this center adopt the spatial navigation terms shown in **Figure** **1**. The structure of FeMo‐co including the homocitrate ligand is also shown in Figure 1: FeV‐co differs slightly by replacement of S3A with a CO_3_ ligand bridging Fe4 and Fe5.

## 4 Overview

I first describe the occurrence and distribution of water molecules in the various crystal structures of protein component 1, extending from the central catalytic site, FeMo‐co or FeV‐co or FeFe‐co, to the surface of the surrounding protein.

### 4.1 Mo Nitrogenases

#### 4.1.1 Av1 Mo Protein

**Figure 2:**
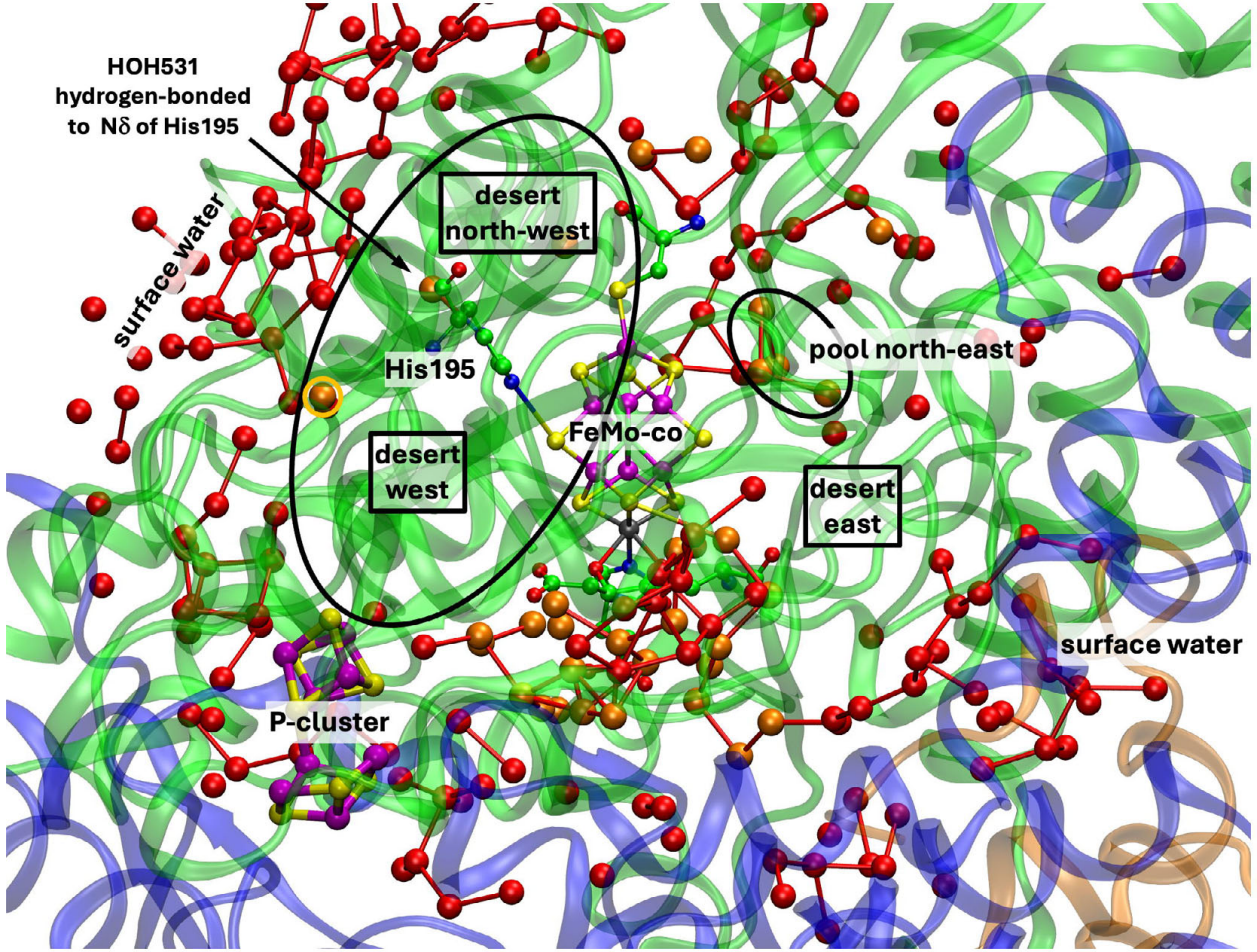
Projection through the *Av*1 protein (PDB 3U7Q) surrounding the FeMo‐co cluster at the center. The view direction is normal to the Fe2‐Fe3‐Fe6‐Fe7 face. Subunit *α* is green, *β* is blue, and *δ* is orange. Red spheres are water molecules 10 to 20 Å from FeMo‐co, and orange spheres are water molecules within 10 Å of FeMo‐co. Probable hydrogen bonds between water molecules (<3.2 Å) are marked with connecting lines. Not all of the water molecules on the surface of the protein are shown. One water molecule orange (circled) is within the oval boundary of desert west and 9.9 Å from S2B.

**Figure** **2** is a projection through an approximately 45 Å diameter sphere of the MoFe protein (*Av*1, PDB entry 3U7Q^[^ ^5^ ^]^) centered on FeMo‐co in the *α* subunit. Atoms of the significant residue His195 and the P‐cluster are included. The water molecules drawn as spheres are color differentiated as orange, within 10 Å of any atom of FeMo‐co, and red which are between 10 and 20 Å from FeMo‐co. The “bonds” drawn between water molecules represent separations < 3.2 Å and probable hydrogen bonds. There is a congregation of close water molecules around homocitrate at the southern part of FeMo‐co. A characteristic set of four waters constitute the small pool to the northeast of FeMo‐co. An important conserved water molecule (here HOH531) is hydrogen bonded to the rear N*δ* atom of the His195 sidechain: the hydrogen bond from His195N*ε* to S2B of FeMo‐co is also marked. The analogous projection through the protein surrounding the other FeMo‐co site (in the *γ* subunit) is identical with that in Figure 2, except of course for the protein surface water.

An outstanding property of this protein is the presence of deserts almost totally devoid of water and adjacent to the cofactor. The oval drawn on Figure 2 defines an oval cylinder that extends from FeMo‐co to surface water at the western edge. The dimensions of this cylindrical prism are ca 9 Å short axis, 16 Å long axis, and depth almost 40 Å. The desert within this volume extends to the west and northwest of FeMo‐co and from the front of the protein to the back. A smaller desert on the eastern side of FeMo‐co is identified on Figure 2. The western desert and the eastern desert marked on the figure are connected behind FeMo‐co, where there is also no water. Note also on Figure 2 that there are no water molecules in front of the cofactor, which apart from the aggregate of four water molecules labeled northeast pool is entirely encircled by desert. The diffraction analysis that generated this crystal structure (PDB 3U7Q) was high resolution (1.00 Å) and defined 2602 water molecules, so it is unlikely that water molecules were overlooked in the analysis and that the deserts are real. They are discussed further in Section 5.

#### 4.1.2 *Cp*1 and *Kp*1 Mo Proteins

**Figure 3:**
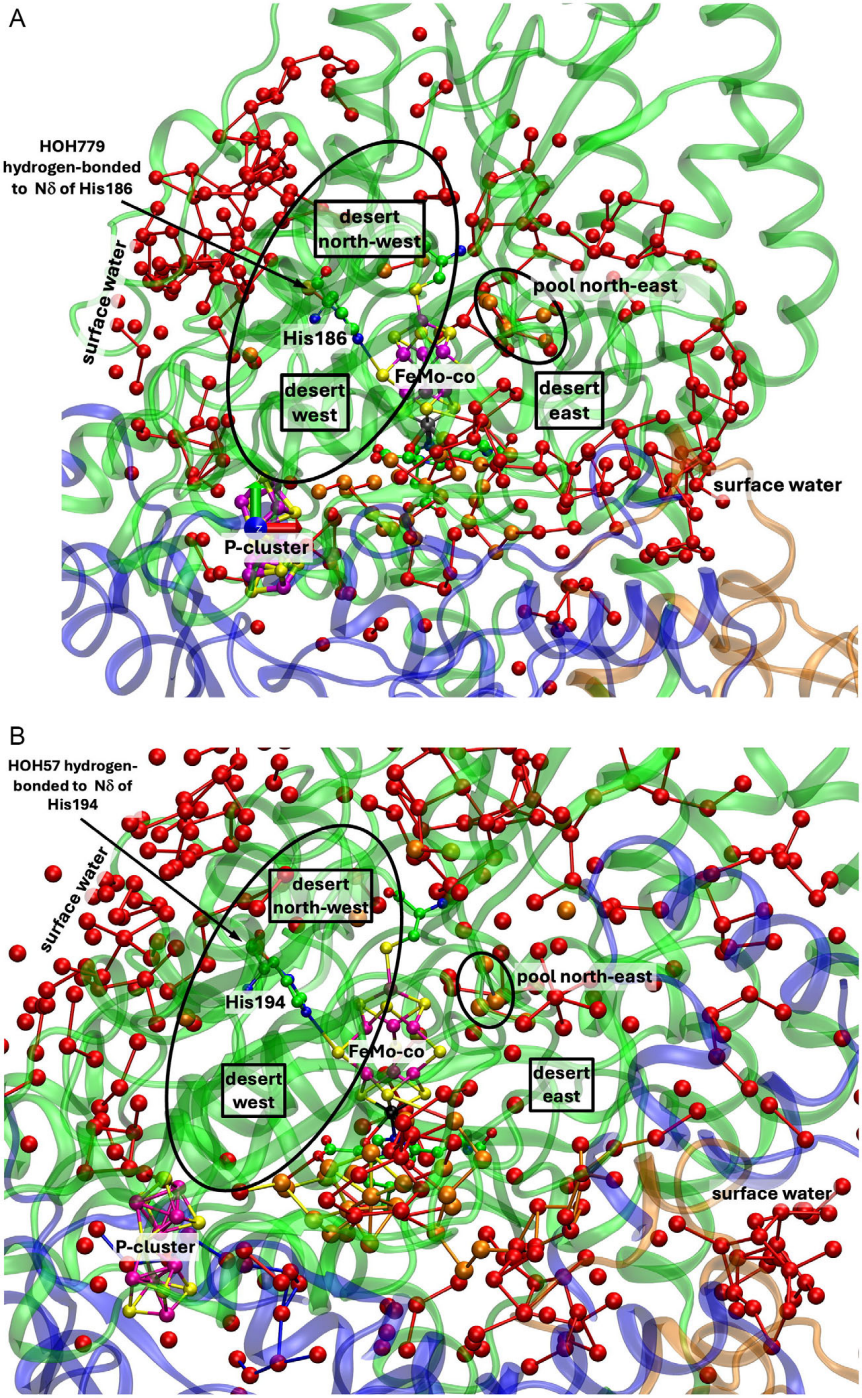
Protein structure in the *α* domains of *Cp*1 A) (PDB 4WES) and *Kp*1 B) (PDB 1QGU), drawn as in Figure 2. Subunit *α* is green, *β* is blue, and *δ* is orange. Spheres colored orange are water molecules within 10 Å of any atom of the FeMo cofactor. The distance limit for red water molecules in **B** (*Kp*1) is increased from 20 to 25 Å, showing additional water on the protein surface. The drawn connections between water molecules are potential hydrogen bonds (<3.2 Å).

**Figure** **3** shows the corresponding projections through MoFe protein in the two other species for which structural information is available. For *Cp*, the structure is PDB 4WES at 1.08 Å resolution and containing 2484 water molecules. The structure for *Kp*1 (reduced form, PDB entry 1QGU) preceded knowledge of the central atom in FeMo‐co but contains 2755 water molecules with diffraction resolution 1.60 Å.^[^ ^30^ ^]^ In Figure 3 B showing the *Kp*1 protein, the inclusion radius was increased to 25 Å from any atom in FeMo‐co in order to demonstrate that the additional red waters (10 to 25 Å from any atom of FeMo‐co) are on the protein surface: the western desert is clear and a few surface waters appear over the eastern desert.

As in the *Av* protein, a single water resides behind His188 (*Cp*) or His194 (*Kp*). The north east pool also occurs but with varied composition and structure (analyzed further in Section 10). The large aggregate of water molecules to the south of FeMo‐co and around homocitrate occurs in all three MoFe protein structures. Differences in the tertiary structures of the *Cp*1, *Kp*1, and *Av*1 proteins are apparent in Figures 2 and 3, but overall the water features are analogous.

### 4.2 V Nitrogenase

**Figure 4:**
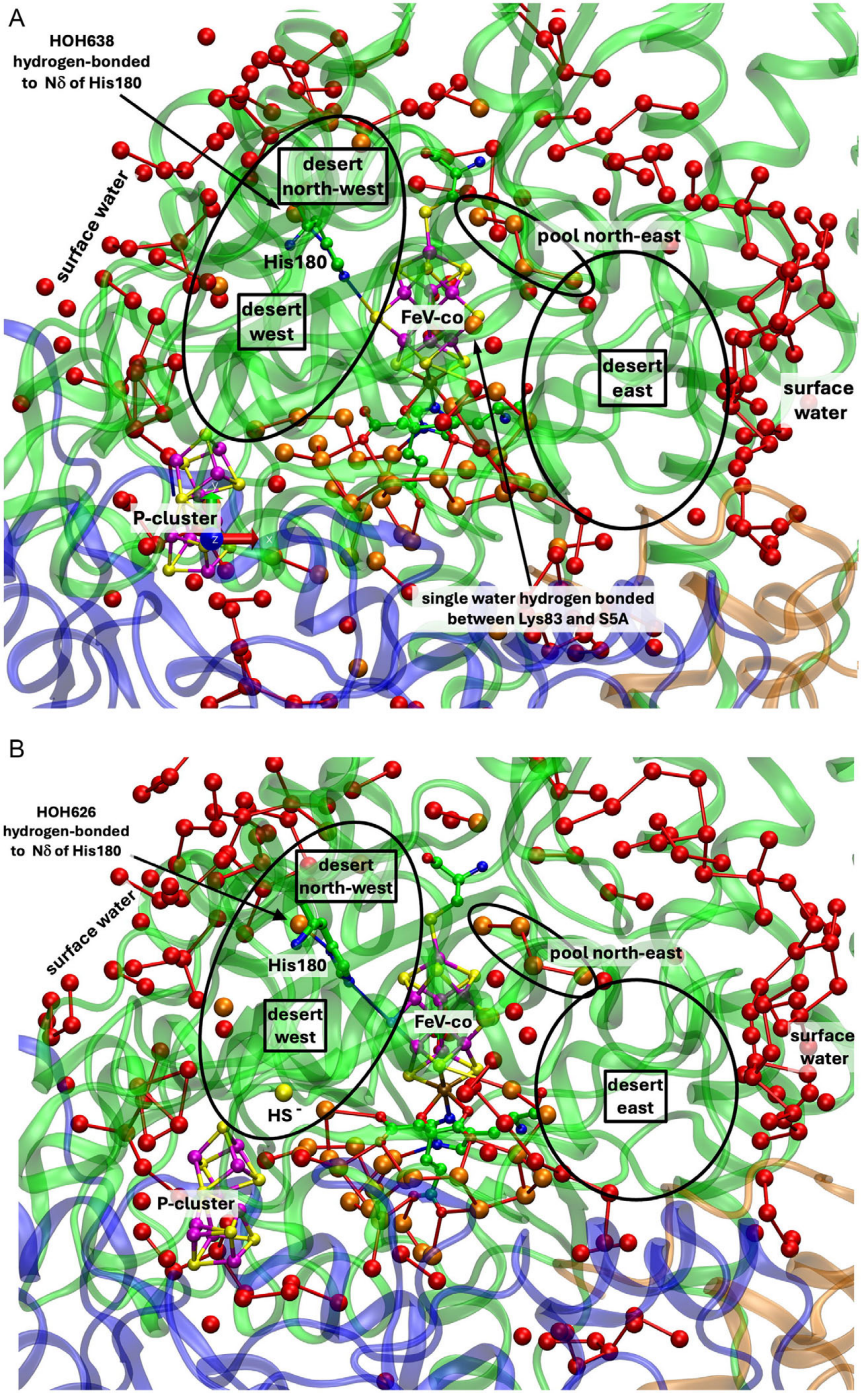
Distribution of water surrounding the FeV‐cofactor in the *α* subunit of the VFe protein of *Av*. Orange spheres are water molecules within 10 Å of the cofactor, and red spheres are waters between 10 and 20 Å distant from the cofactor. His180 in the *Av* VFe protein corresponds to His195 in the *Av* MoFe protein. Subunit *α* is green, *β* blue, and *ε* orange. A) The resting state, PDB 5N6Y.^[^ ^31^ ^]^ B) A putative turnover state, PDB 6FEA,^[^ ^32^ ^]^ with S2B displaced as HS^‐^, marked as a yellow sphere in the lower section of desert west.

**Figure** **4** shows the analogous tertiary protein and water structure in two forms of the *Av* protein containing the cofactor FeV‐co. The crystal structures are the resting state, PDB entry 5N6Y (resolution 1.35 Å, 2249 waters),^[^ ^31^ ^]^ and the same enzyme in a putative turnover state, PDB entry 6FEA (resolution 1.2 Å, 2652 waters).^[^ ^32^ ^]^ The cofactor FeV‐co contains a bridging CO_3_ ligand (carbonate or hydrogen carbonate) in place of S3A on the back side FeV‐co. The *Av* VFe proteins also differ from the Mo counterparts at S5A. The guanidine side chain of Arg96 hydrogen bonds directly to S5A in the Mo proteins, while in the V proteins, the corresponding residue is Lys83, and a single water molecule hydrogen bonds the Lys‐NH_2_ terminus to S5A. Crystal 6FEA is reported to have captured a turnover state of the enzyme, in which the bridging atom S2B of FeV‐co is displaced by 7 Å and resides in a pocket as HS^‐^.^[^ ^32^ ^]^ This position is marked on Figure 4 B. This crystal also contains an alternative conformation for a nearby glutamine residue, not evident in the present pictorial presentation.

The view direction and the pictorial parameters are the same in Figures 4 and 2, allowing direct comparison of the VFe and MoFe proteins. Both possess the extensive desert on the west side. On the east side of the FeV cofactor, the desert (circled) is clearly defined and extends further south than the comparable east desert in the *Av* MoFe protein. There is a distinctive congregation of surface water molecules east and northeast of the eastern desert in the two VFe proteins: these are almost 20 Å from the cofactor. This surface water feature does not appear as such in the MoFe proteins.

### 4.3 Fe Nitrogenase

**Figure 5:**
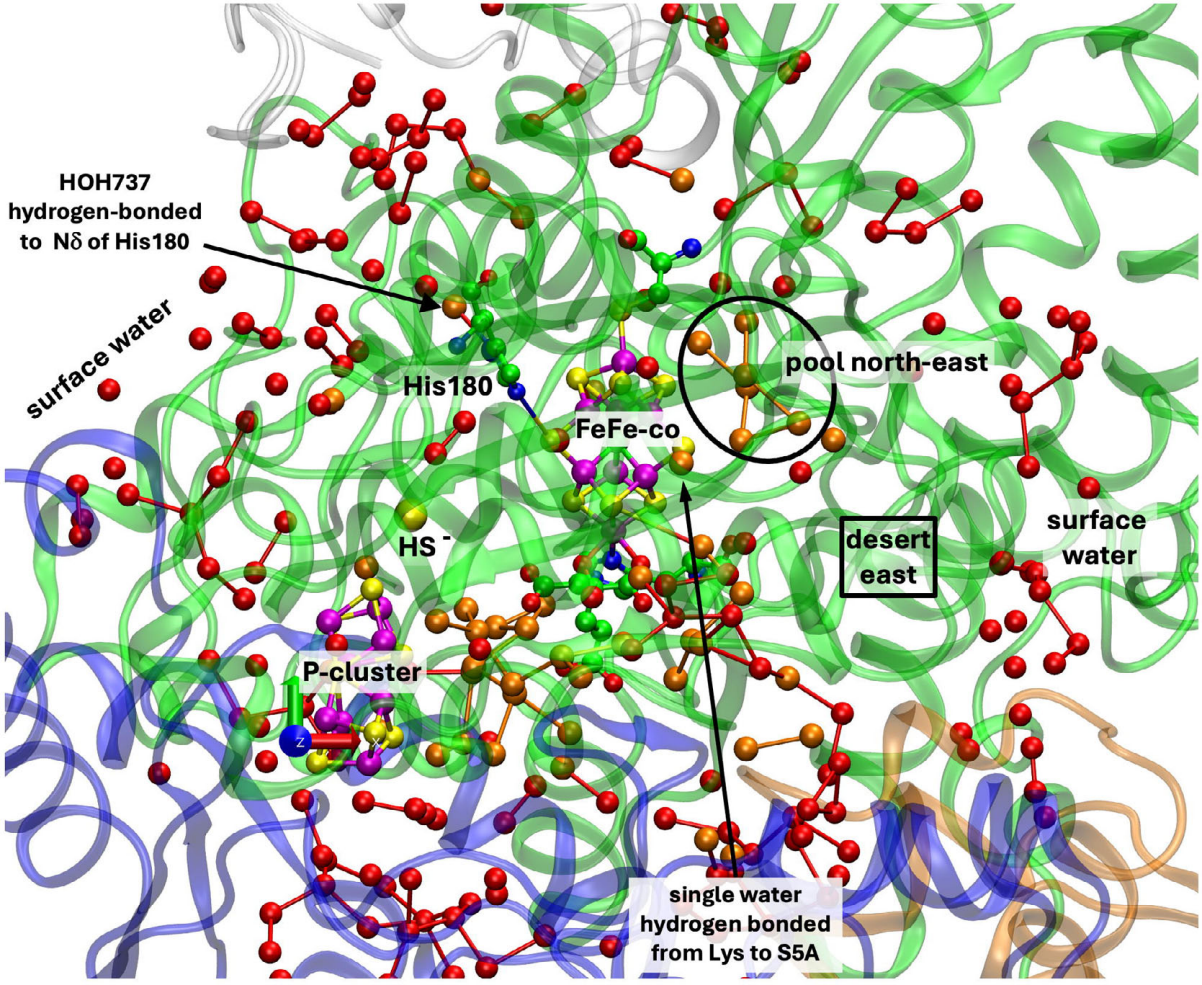
The protein and water structure around FeFe‐co in the *Av*1 protein crystal PDB 8BOQ. Subunit *α* is green, *β* blue, *γ* white, and *ε* orange. Orange spheres are water molecules within 10 Å of the cofactor, and red spheres are waters between 10 and 20 Å distant from the cofactor. HS^‐^ is S2B partially displaced from FeFe‐co.

The one crystal structure of the FeFe protein (*Av*1, PDB 8BOQ) is described as a mixture of a resting state and a nonresting state and contains an HS^‐^ entity as S2B displaced from the Fe cofactor.^[^ ^33^ ^]^ The number of water molecules resolved in this crystal structure (1428) is less than for the Mo and V proteins (2249 to 2735 waters), and so conclusions about water structure are less certain. **Figure** **5** shows the protein and water structure around FeFe‐co in the *α* subunit. Western and eastern deserts are apparent. The water behind His180 and the northeastern pool are present, but there are fewer water molecules in the surface water domains. As in the VFe proteins, there is a Lys‐water‐S5A hydrogen bonding connection.

In summary at this point, all crystal structures for the Mo, V, and Fe component 1 proteins contain extensive and deep deserts to the west and east of the cofactor, with variations. All proteins contain a pool northeast of the cluster, also with variations in composition and arrangement. All proteins contain a lone water molecule hydrogen bonded to N*δ* of the histidine residue that is also hydrogen bonded by N*ε* to S2B of the cofactor. All proteins have an aggregate of many water molecules near homocitrate and to the south and southwest the cofactor, also with substantial variations in composition and structure. Each of these common features is described and analyzed further in the following sections.

## 5 Deserts

**Figure 6:**
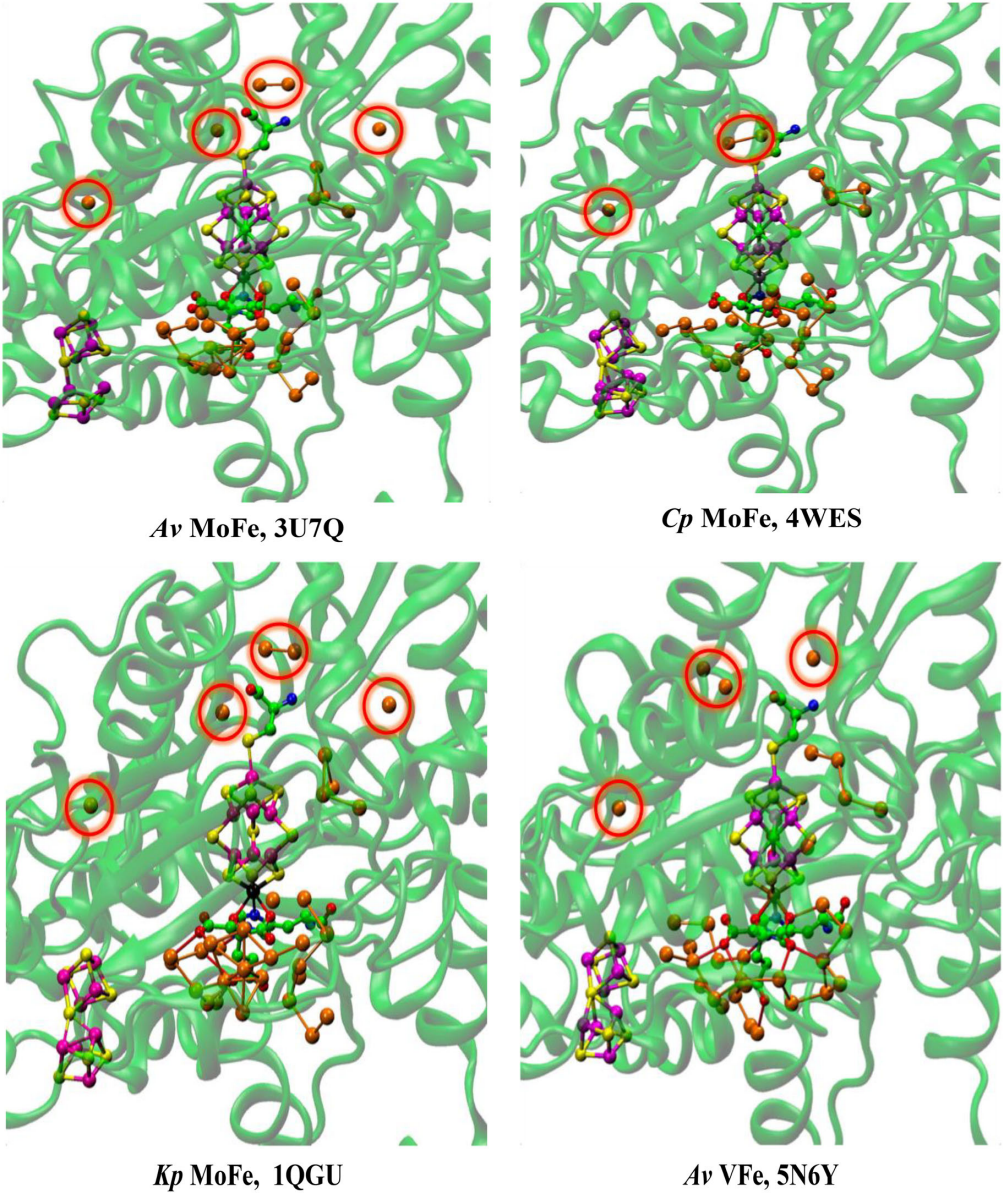
Comparative images of the desert regions in the MoFe and VFe proteins, all in the *α* subunit. The cofactor at the center is oriented front‐on in all images. The northeast pool and the southern water aggregates around homocitrate are not excluded. Water molecules in the desert regions and within 10 Å of any atom in the cofactor are emphasized with red circles.

The deserts identified above possess significant properties that are examined here, concentrating on the MoFe proteins of the three species and the *Av* VFe protein. **Figure** **6** shows comparative pictures of these four structures, with identical orientations about the cofactor axes, and simplified by removal of the surface waters and of the single water plus histidine hydrogen bonded to S2B. The small water pool to the northeast and the southern water pool around homocitrate are retained in these images. The tertiary structure of only one subunit is drawn, because the cofactor and its surrounding desert are located within this one subunit. Water molecules that reside in the desert and are within 10 Å of any part of the cofactor are highlighted with red circles. There are five such waters (oases?) in the *Av* and *Kp* MoFe proteins, three in the *Cp* MoFe protein, and four in the *Av* VFe protein.

The locations of these waters relative to the cofactor are similar. The single water near the western protein surface is almost identical in all four structures, but the pair of waters to the north have distances from the cofactor varying by 3 Å. Distances of these waters from the center of the cofactor are 12.1, 12.5, 12.2, and 12.6 Å in 3U7Q; 11.9, 8.7, and 9.2 Å in 4WES; and 11.8, 11.4, 12.0, and 12.6 Å in 5N6Y. Close examination of the green ribbons shows that these northern waters have different protein cages. Note that water in the desert is very similar in the *Av* and *Kp* MoFe proteins.

The images in Figure 6 also demonstrate how the desert extends in most directions around the cofactor: only the south and the northeast of the cofactor are not exposed to desert regions. The west, east, front, and back of the cofactor are devoid of water. It is particularly significant that the catalytic reaction zone of the cofactor, near Fe2 and Fe6 on its western edge, is totally dry. This has mechanistic implications: water cannot be invoked here as a protonation agent for bound N_2_ or for subsequent bound hydrogenated forms of N_2_.

Are these deserts unusually dry? Carugo has reviewed and examined the occurrence of water buried inside proteins, using high‐quality crystal structures from the Protein Data Bank.^[^ ^34^ ^,^ ^35^ ^]^ He defines lake‐like structures containing one or two waters and found that on average, there is one buried water molecule in lake‐like clusters per 36 residues. More residues per buried water molecule (48) are observed in all‐alpha proteins and fewer (32) in all‐beta proteins. Alpha–beta proteins have one buried water molecule per 37 residues. Application of these statistics to the deserts described here in the nitrogenase proteins is at best semiquantitative, because the extent of the deserts and the number of residues contained are ill‐defined. The region enclosed by the domain 10 Å from any atom of the cofactor is roughly 50% of the subunit, and the space occupied by the southern water pools is about 20% of the subunit, so I estimate that the space in the protein classified as desert is about 30% of the subunit. The *Av* MoFe *α* subunit in crystal 3U7Q contains 477 residues (VFe in crystal 5N6Y contains 474 residues), so the desert regions contain about 140 residues. The MoFe and VFe *α* subunits are predominantly alpha‐conformed (56% of residues). Application of the Carugo statistic of one water per 48 residues translates to ca 3 (140/48) buried waters per desert region. It seems that the desert regions in the nitrogenase proteins are not unusually dry.

The significance of the deserts is their location, particularly at the catalytic reaction zone near Fe2 and Fe6, where multiple replenishable protons are required. This requirement focuses attention on the water‐rich domains: I now turn to the rivers in the nitrogenase proteins.

## 6 Rivers

The MoFe and VFe proteins contain rivers—continuous sequences of water molecules—connected to the catalytic cofactors. The following descriptions exclude the one crystal structure of the FeFe protein because it has a deficiency of resolved water molecules, with uncertain remnants of the water chains. There are substantial differences between the water chains of the MoFe and VFe proteins.

### 6.1 Mo Nitrogenase

**Figure 7:**
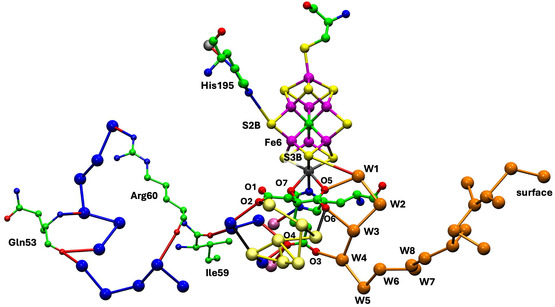
Water paths connected to the FeMo cofactor in the *α* subunit of the *Av* MoFe protein (PDB 3U7Q). Water molecules colored orange constitute the water path leading from near the protein surface on the right to atoms O3, O6, and O5 of homocitrate and then to S3B of FeMo‐co.^[^ ^21^ ^,^ ^23^ ^]^ Water molecules colored blue are associated with the pathway for egress of product NH_3_ to the left.^[^ ^22^ ^]^ The water molecules colored yellow are part of an irregular pool near homocitrate. There is an isolated lilac water hydrogen bonded to N*ε* of His442, the ligand for Mo.

**Figure** **7** shows two winding rivers—color‐coded orange and blue—that connect to FeMo‐co in the *Av*1 protein. The orange waters constitute a continuous chain linking the surface of the protein to S3B of the cofactor. There are hydrogen bond connections to three carboxylate oxygens (O5, O6, and O3) of homocitrate. This fully hydrogen bonded water chain has been described previously, as the chain that supplies serially the protons need for catalysis at the cofactor.^[^ ^21^ ^,^ ^23^ ^]^ This crucial functionality of the orange river and the Grotthuss mechanism by which it operates are described in Section 8.1 and 8.2. The orange river passes to the south of the eastern desert (see Figure 2), and in the *α* subunit has hydrogen bonding connections mainly to the A and D polypeptide chains.

The sequence of water molecules colored blue in Figure 7 follows the proposed pathway for the passage of product NH_3_ to the external medium, after dissociation from the catalytic cofactor. NH_3_ is translocated by skipping through a sequence of hydrogen bonds involving eleven water molecules and surrounding amino acids.^[^ ^22^ ^]^ The residues that link disconnected sections of this water chain are marked on Figure 7. The blue “ammonia river” (with “waterfalls”) is located at the southern end of the western desert on Figure 2 and passes behind the P‐cluster. Further details of the river that transports ammonia are presented in Section 9.2.

It is significant that the opposite directions of the protonic water chain to the east and the ammonia chain to the west embody the separation of the acidic reactant and the basic product of the nitrogenase reaction. Protonation of product NH_3_ would both waste an incoming proton and, as elaborated in Section 9.2, reduce the mobility of departing NH_3_.

### 6.2 V Nitrogenase

**Figure 8:**
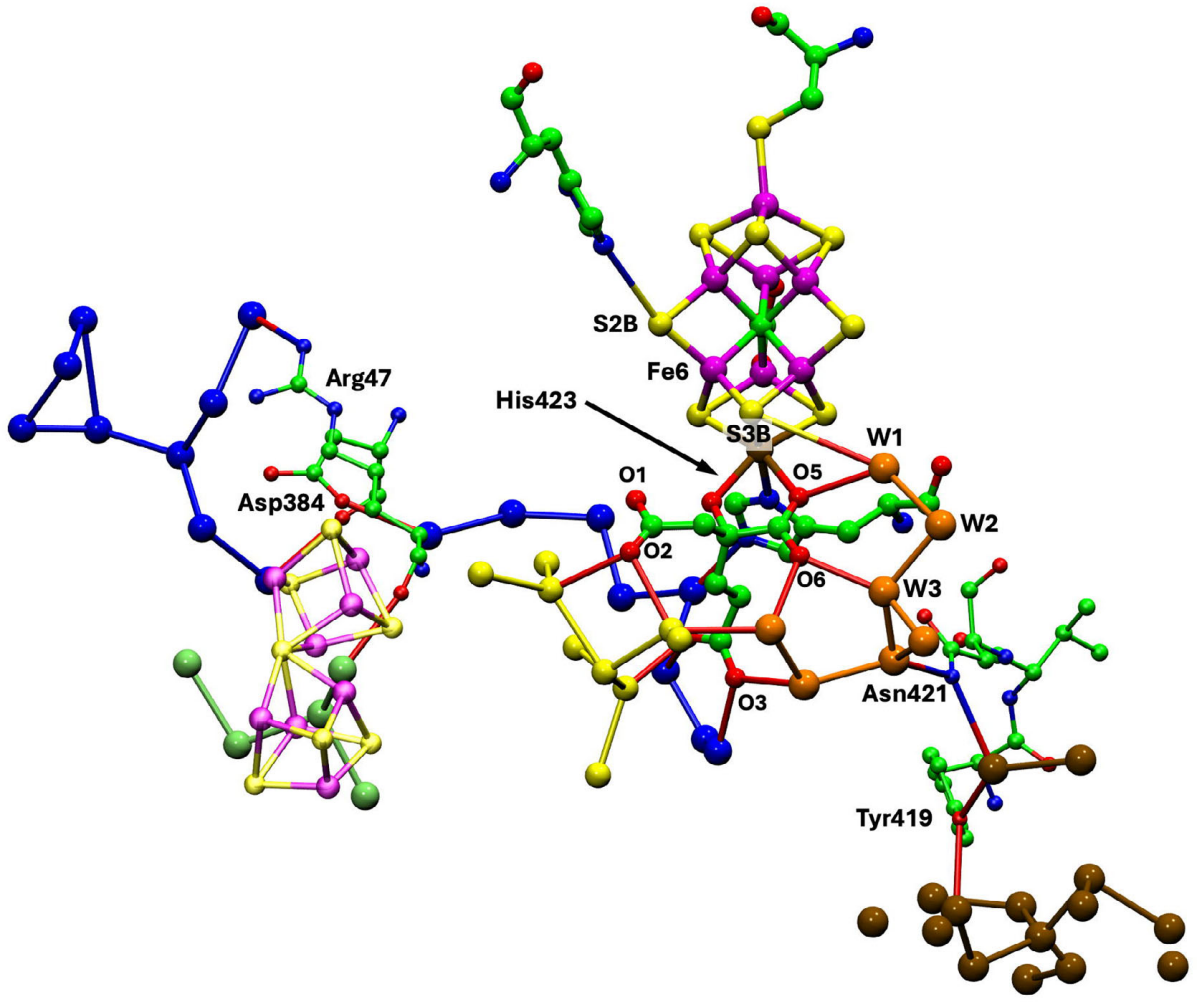
Water paths in the *δ* subunit of the VFe protein of *Av* vanadium nitrogenase (PDB 5N6Y). Orange W1, W2, and W3 are the same as in the MoFe protein, but the other four orange waters are different. The water chain colored blue is hydrogen bonded to N*ε* of His423, shares O3 of homocitrate with the orange path, and passes behind the P‐cluster. The gap in the blue chain is bridged by O and sidechain carboxylate of Asp384. Arg47 is connected to the blue river only at its sidechain, while the O of Arg47 hydrogen bonds to an isolated aggregate of five waters (lime) behind the P‐cluster.

**Figure** **8** shows the water structure in the region below the FeV cofactor in the *δ* subunit of the VFe protein of *Av* vanadium nitrogenase (PDB 5N6Y): the other subunit (*α*) is very similar. This water structure has significant differences from that of Mo nitrogenase, evident in side‐by‐side comparison of Figures 7 and 8. Note specially the absence of the orange water chain constituting the proton wire in the Mo protein. The first three waters, W1, W2, and W3 (orange), and their engagement with O5 and O6 of homocitrate are the same for V and Mo, but then the orange chain bifurcates with additional water before hydrogen bonding to homocitrate O3 (compare Figure 7 and 8). There is a hydrogen bond connection to the yellow water aggregate, which does not occur in the Mo proteins. South of the orange waters there is a waterless gap to a pair of waters (brown) and then another gap to a large group of brown water molecules. Hydrogen bonds from the NH_2_ group on the sidechain of Asn421 connect an orange water to one of the pair of brown waters, which is further connected via hydrogen bonds using the OH group of Tyr419 to the more southern brown group. Further detail of the brown waters in the VFe protein is developed in Section 8.3.

In the VFe protein there is a chain of water molecules, colored blue, separated from the yellow aggregate, and partially analogous to the blue chain of the MoFe protein. In both proteins, these rivers pass behind the P‐cluster. In MoFe, the blue and orange chains have separate connections to the long arm carboxylate of homocitrate, blue to O4 and orange to O3. However, in VFe, both chains hydrogen bond to O3. The blue chain in VFe protein forms a hydrogen bond with N*ε* of His423, which is the histidine that coordinates to V via N*δ*.

**Figure 9:**
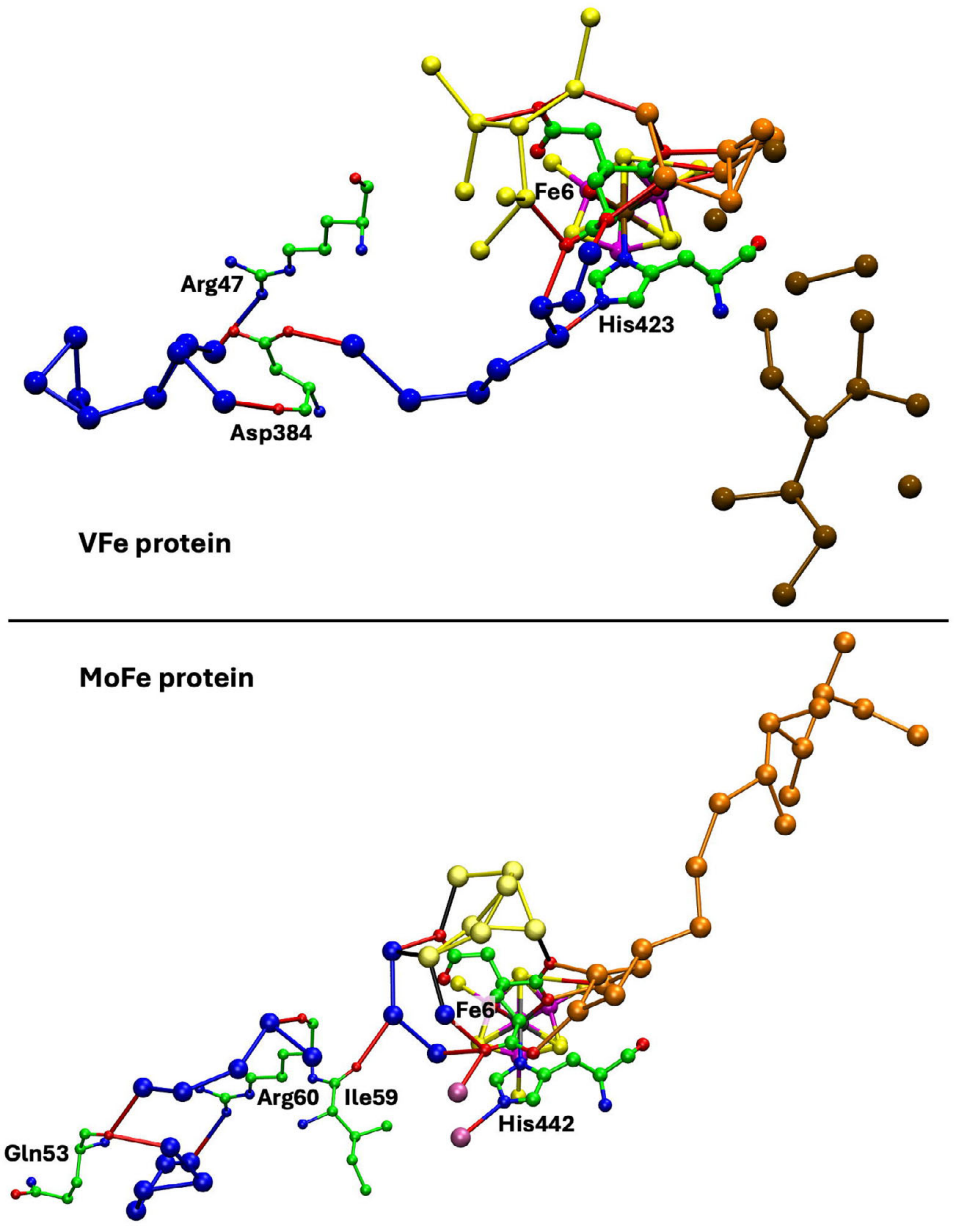
Comparison of the water paths in the VFe protein (*δ* subunit, PDB 5N6Y) and the MoFe protein (*α* subunit, PDB 3U7Q). The same view direction in both is along the pseudo‐three‐fold axis of the cofactor, with the homocitrate end nearer. The CO_3_ ligand on FeV‐co is deleted to improve clarity. Water paths are colored as in Figures 7 and 8. The histidine ligating V or Mo is labeled, to draw attention to the different water hydrogen bonds with N*ε*.

The water architecture of the VFe protein and its difference with that of the MoFe protein is further emphasized in **Figure** **9**. The view direction is along the pseudo‐threefold axis of the cofactor, looking towards the homocitrate end: this view is orthogonal to that in Figure 8. The filamented aggregate of brown water molecules in the VFe protein is outstandingly different in both structure and direction from the orange water chain in the MoFe protein. There is no similarity. The direction of the sequence of blue waters in VFe protein has some similarities with that in the MoFe protein, but there are distinct differences. In VFe, the blue chain is hydrogen bonded to N*ε* of the histidine ligand (423), whereas in MoFe, the water (lilac) hydrogen bonded to N*ε* of His442 is separate from the blue chain, which connects to a different region of the cofactor and homocitrate. The blue chain interruption occurs further along in the VFe protein, and the arginine residue bridges the interruption differently in the V and Mo proteins. Note also that the view of the MoFe protein in Figure 9 clearly shows the opposite directions of the proton wire water path and the ammonia path.

**Figure 10:**
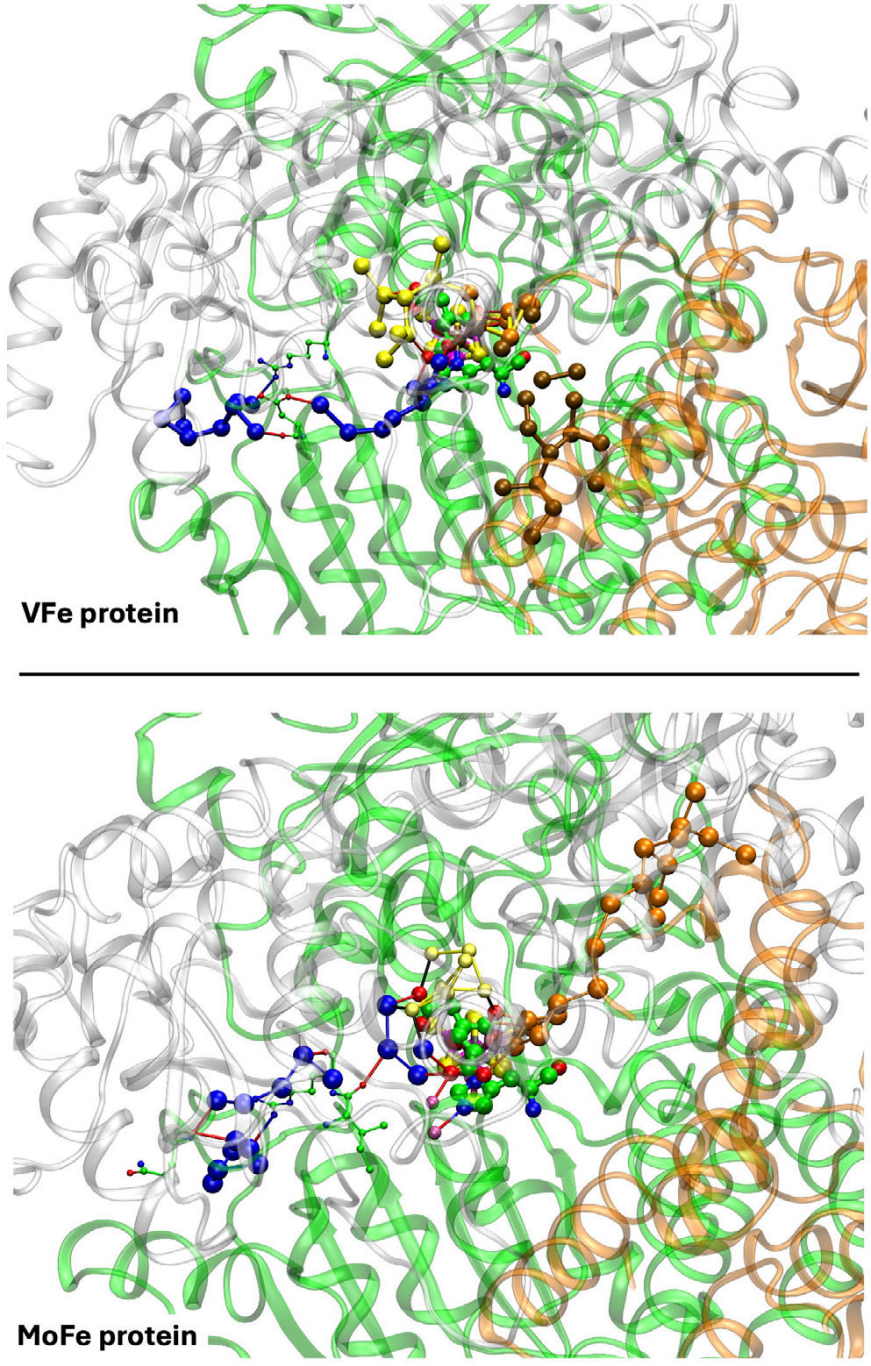
Comparison of the protein structure around the cofactor and the water paths in the VFe protein (PDB 5N6Y, *δ* subunit green, *β* subunit white, *ε* subunit orange) and the MoFe protein (PDB 3U7Q, *α* subunit green, *β* subunit white, *δ* subunit orange). The view direction is the same as that of Figure 9.

One question is whether differences in the secondary and tertiary structure of the V and Mo proteins influence their differences in water structure. Is different protein structure the reason for the VFe protein not using the well‐defined orange water chain of the MoFe protein? The overall structure of the V and Mo proteins has been described: the four comparable subunits in the two structures (PDB 5N6Y and 3U7Q) are aligned with a rms deviation of 1.97 Å for all atoms.^[^ ^31^ ^]^ **Figure** **10** is a pictorial comparison of protein structure in relation to the river water structure. The similarities and small differences in protein structure are apparent. Although this pictorial analysis is coarse grained, there is no evident reason why the VFe protein does not support a well‐formed proton‐wire water chain, as in the MoFe protein.

### 6.3 Water Connections to Histidine Ligand

The VFe proteins shown in Figure 8 and 9 reveal the chain of eight hydrogen bonded blue waters connected to the N*ε*H group of His423, which also ligates vanadium via N*δ*. The MoFe proteins are distinctly different, all having just one water receiving a hydrogen bond from N*ε*H of the ligating histidine. This water is evident (lilac coloration) in Figure 7 and 9. In all MoFe proterins, this water is also hydrogen bonded to the side chain of a glutamate residue (380 in *Av*, 467 in *Cp*, 425 in *Kp*).

## 7 Homocitrate Pool

I now describe the collection of water molecules around homocitrate, colored yellow in pictures above, and located between the blue river and the orange river. Are these yellow waters connected to the orange and blue rivers? How extensive is the yellow aggregate, and how is it structured? Are these structures conserved? There are differences between the Mo and V proteins, and I describe first the yellow waters in the MoFe proteins of the three species *Av*, *Cp*, and *Kp*.

### 7.1 Mo Nitrogenase

**Figure 11:**
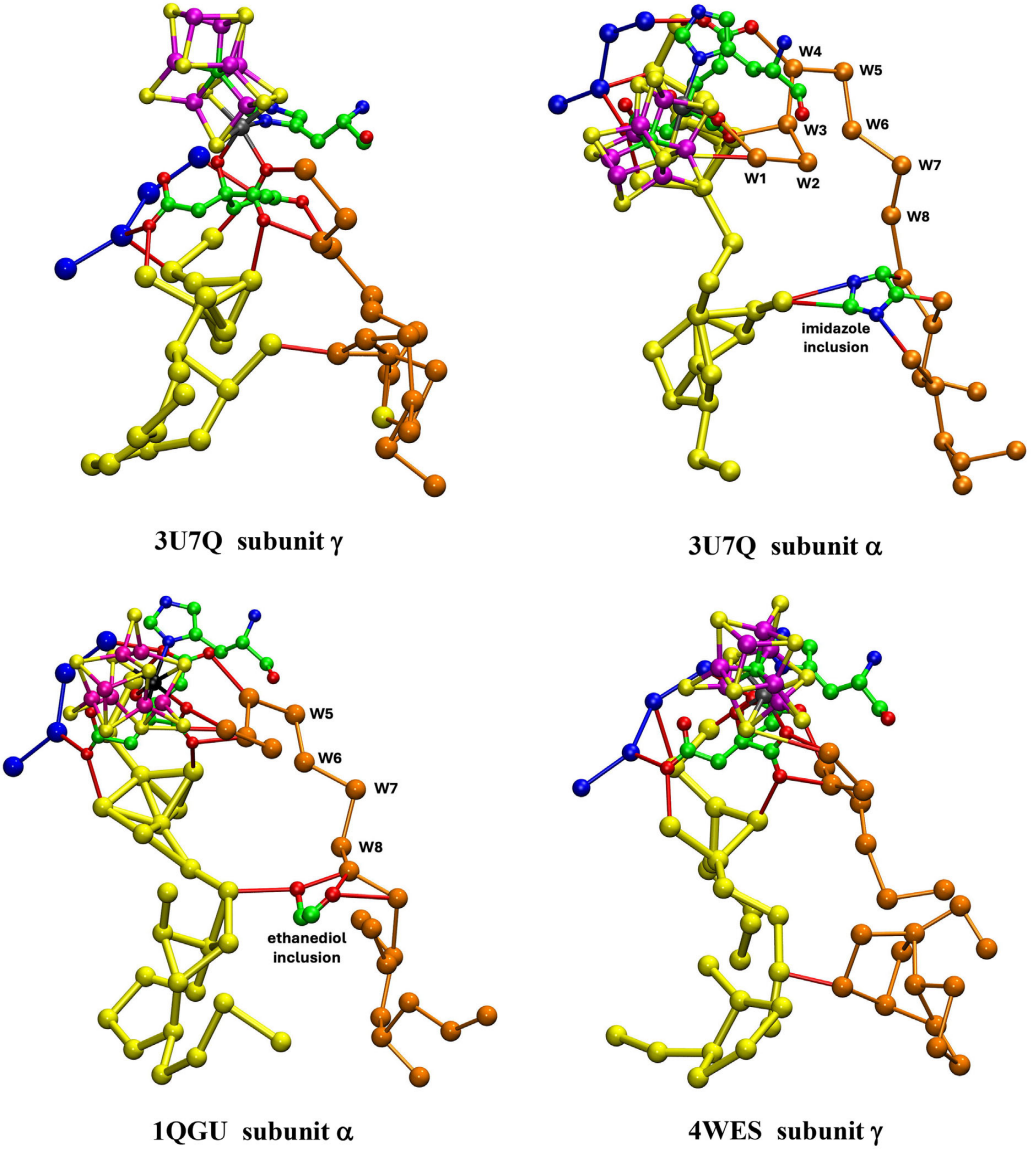
The water aggregates, yellow, that extend from homocitrate, and their relationships with the orange water chains, in four subunits of the three MoFe proteins. Four conserved waters of the blue river are included. Distances < 3.2 Å between water molecules are drawn as bonds. In the *α* subunit of *Av* (3U7Q), there is an imidazole inclusion, with one possible hydrogen bond, and other water contacts that are too short or stereochemically impossible. An ethane‐1,2‐diol inclusion in the *α* subunit of *Kp* forms normal hydrogen bonds with yellow and orange water molecules.

**Figure** **11** pictures four of the six occurrences of the yellow water collections in the two subunits each of the three MoFe proteins. The orange water chains and close components of the blue waters are included. Two obvious properties of the yellow water aggregates are the geometrical variety and the geometrical irregularity. The yellow “bonds” drawn between waters are distances < 3.2 Å, possible hydrogen bonds, but many of these distances occur in triangles with angles ca 60°, very different from the near tetrahedral stereochemistry associated with hydrogen bonded water molecules. Of the six occurrences of the yellow aggregate in the crystals 3U7Q, 4WES, and 1QGU, two of the PDB files contain disordered water molecules or waters in too close contact. Clearly, the yellow aggregates are not orderly hydrogen bonded networks.

The yellow aggregates also have different relationships with the variable sections of the orange chains, beyond the conserved section up to W8. In four of the six occurrences, there is one contact < 3.2 Å between a yellow water and an orange water in this region. In the *α* subunit of *Av*1, an inclusion of imidazole (from the crystallization buffer) occurs between the yellow and orange chains in the variable section beyond W8: this imidazole has one unreal C‐‐O short contact with water (see Figure 11 3U7Q subunit *α*). In the *α* subunit of *Kp*1, ethane‐1,2‐diol is an exogenous inclusion that has normal hydrogen bonds with one water in the yellow chain and with two waters beyond W8 in the orange chain. Both inclusions occur in regions where the water structures are variable in both the orange and yellow chains, which is consistent with these regions being accessible to small molecules from the protein surface and being structurally adjustable for different inclusions.

**Figure 12:**
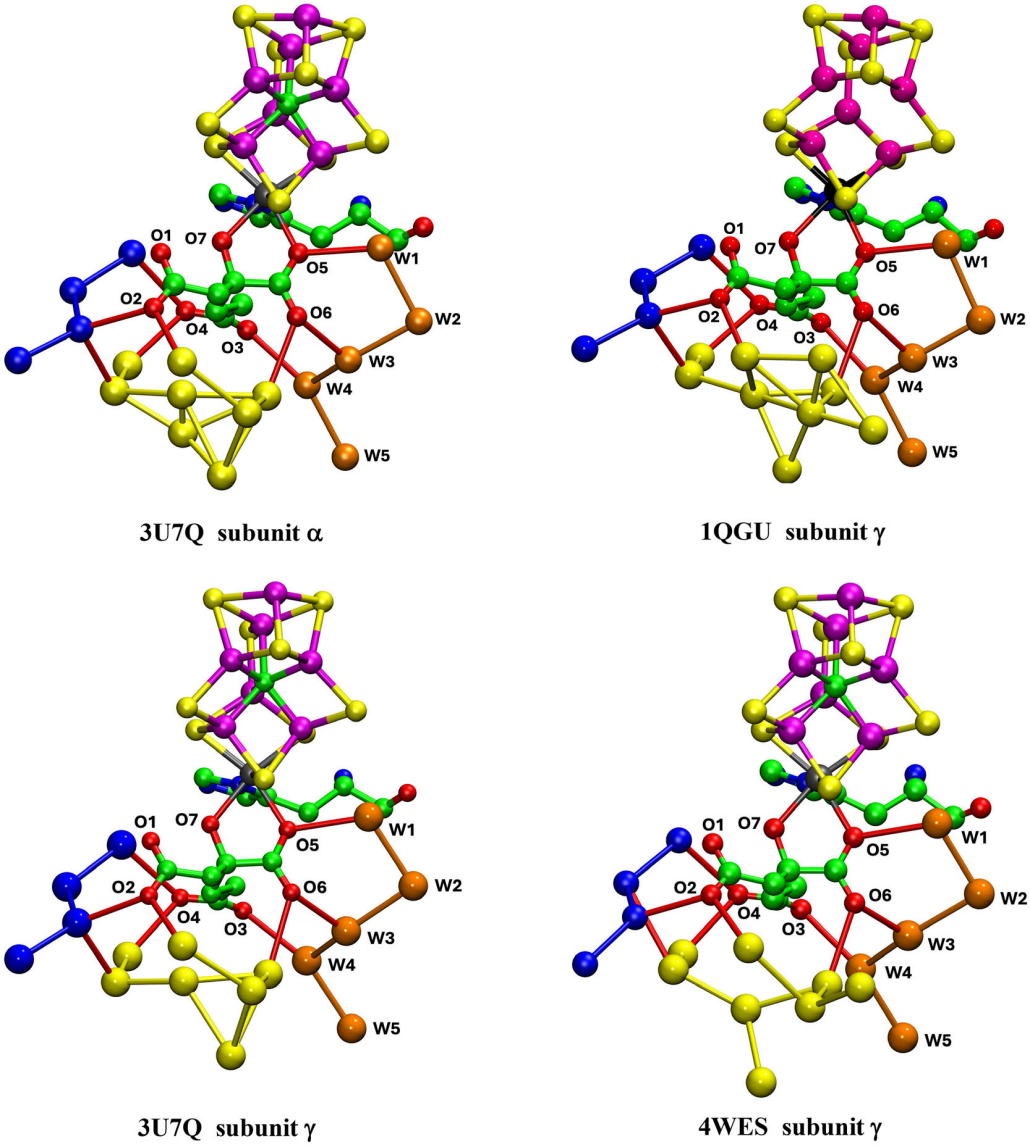
Waters connected to carboxylate O atoms of homocitrate in MoFe proteins. Connections < 3.2 Å are drawn. Two yellow waters in 3U7Q subunit *α* are too close, 1.69 Å.

Figure 11 shows complete yellow and orange aggregates, but obscures most of the connections with homocitrate, details of which are presented in **Figure** **12**. Four unique structures are shown, to illustrate the similarities and differences. Interatomic distances < 3.2 Å and possible hydrogen bonds are drawn. In all cases, (a) O6 of HCA has H bonding contacts with W3 and one yellow water, (b) O3 connects only with W4, (c) there is no direct hydrogen bond between a yellow water and an orange water, (d) O4 connects with one yellow and one blue water, and (e) O2 also connects with one yellow and one blue water. These connections (a), (b), and (d) are stereochemically suited to hydrogen bonding. There is one yellow–blue contact in all proteins. The yellow aggregates in the four instances shown are all different and contain many interatomic angles of ca 60°, unsuited to hydrogen bonding. Again, it is clear that there is no ordered hydrogen bonding network within these yellow water pools close to homocitrate: some of these waters appear to be fluid.

### 7.2 V Nitrogenase

**Figure 13:**
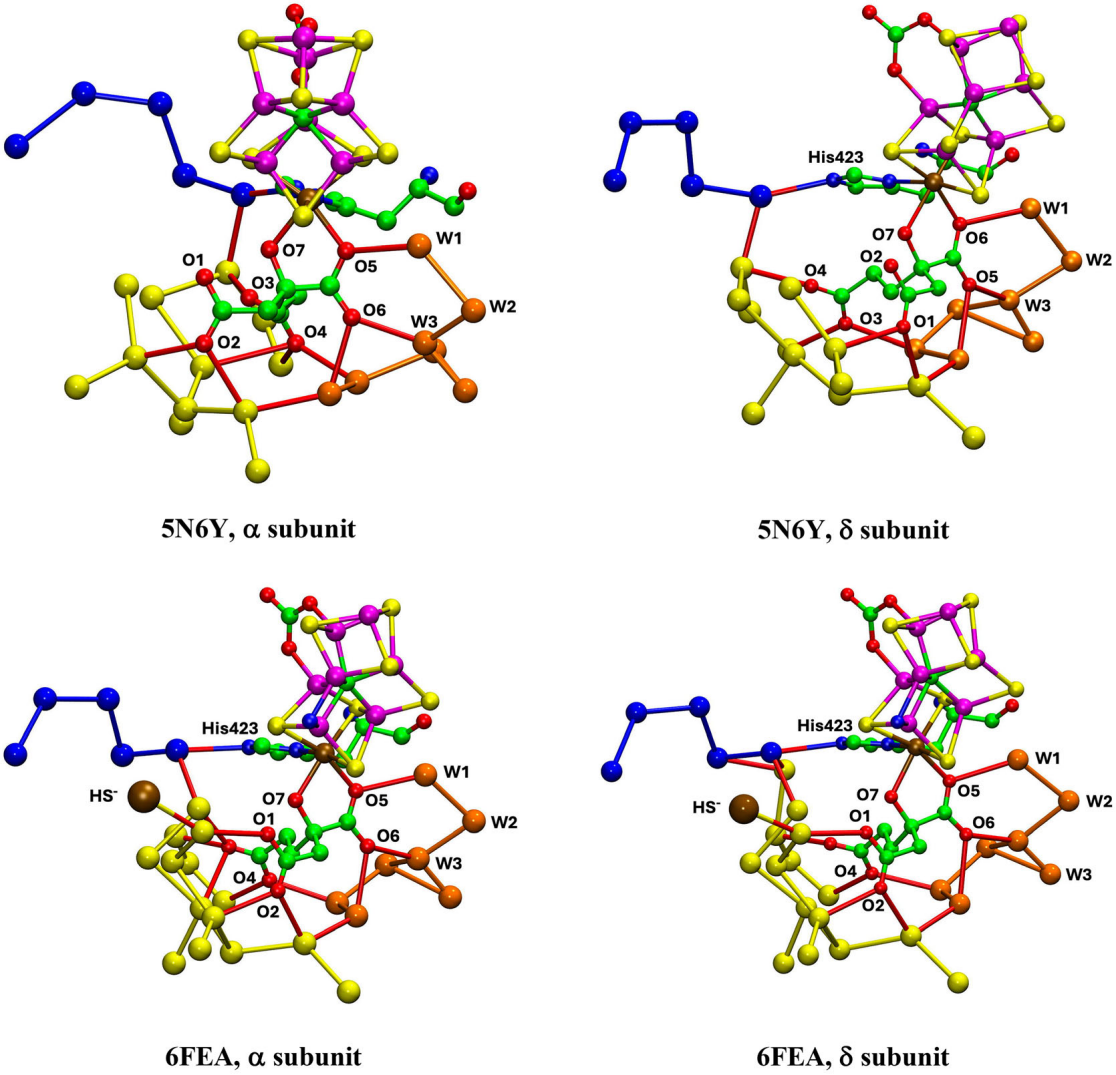
Yellow, orange, and blue water sequences bonded to homocitrate in the VFe protein structures. Upper pictures are the two occurrences in the resting state *Av*1 protein (PDB 5N6Y). The lower pictures (PDB 6FEA) are the two occurrences in the nonresting state of the *Av*1 protein: the ochre sphere is displaced HS^‐^. The homocitrate O atoms in the VFe proteins are labeled as in the PDB files, which is slightly different from that of the MoFe proteins, interchanging O3 and O4 on the long carboxylate arm and O1 and O2 on the short arm.

**Figure** **13** shows the orange, yellow, and blue collections of water molecules connected to and around the O atoms of homocitrate in the crystal structures 5N6Y (resting state) and 6FEA (nonresting state) of the *Av*1 VFe protein. The two occurrences of the homocitrate pool in 5N6Y are similar and contain fewer yellow waters than the two similar pools in 6FEA. These VFe structures are notably different from the corresponding water structures of the MoFe proteins. In the VFe proteins, the yellow aggregate is compact around homocitrate and does not extend away in a yellow river as in the MoFe proteins: note the contrast between the yellow sections of Figure 11 and 13. This difference occurs also in the orange water aggregates, which extend away adjacent to the yellow aggregates in the MoFe proteins but are limited in extent in the VFe proteins. In the MoFe proteins, the orange and yellow aggregates close to homocitrate are not directly hydrogen bonded to each other and are linked only through the separate hydrogen bonds with O6 of homocitrate (Figure 12), but in the VFe proteins there is different orange architecture allowing a direct orange‐yellow hydrogen bond. In all MoFe and VFe structures, there is a direct hydrogen bond between one blue and one yellow water molecule. Homocitrate O atoms are labeled slightly differently in the V and Mo protein structures, but in both, O3 and O4 are on the long arm and O1 and O2 on the short arm.

**Figure 14:**
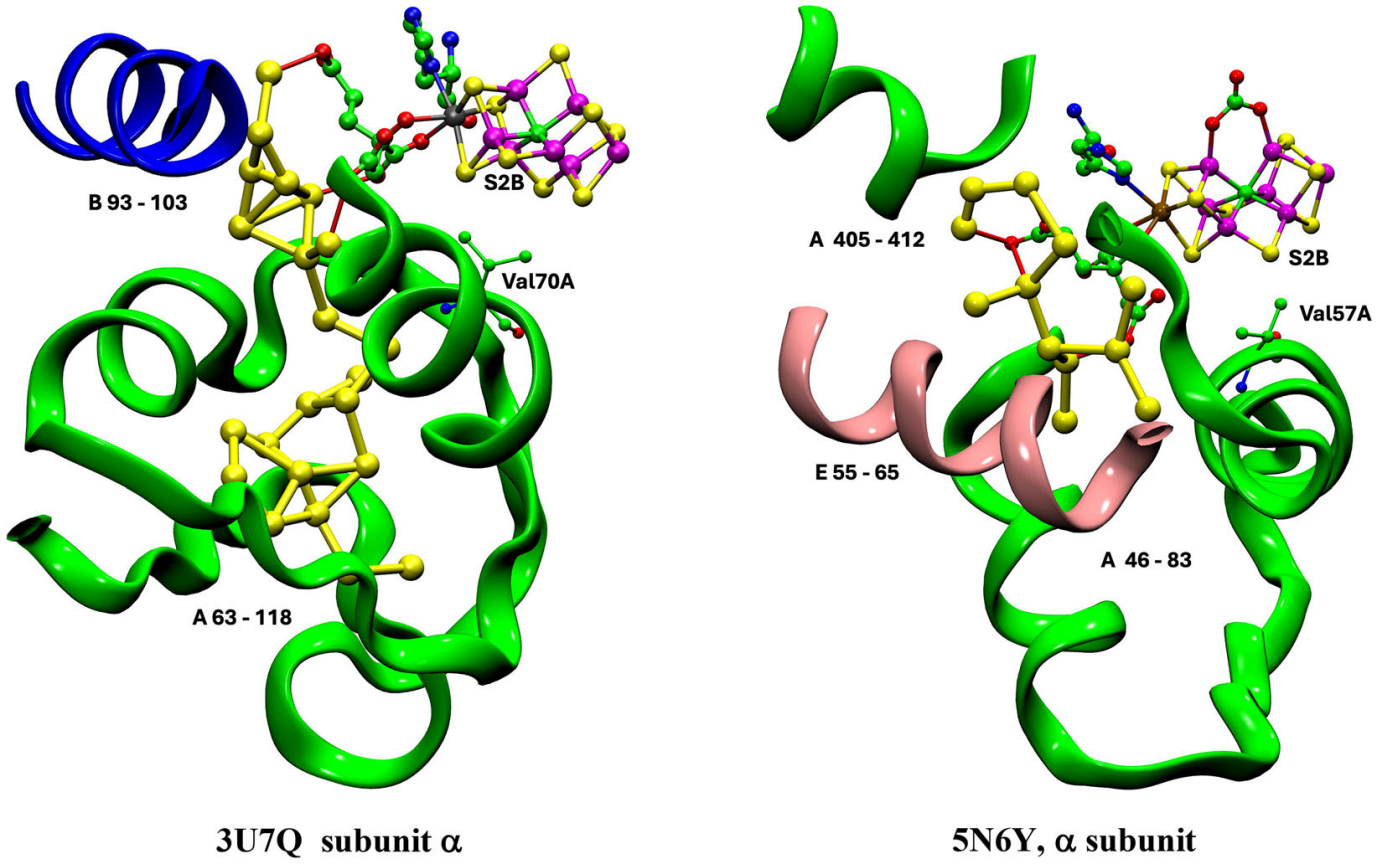
Protein structure around the yellow water aggregate in MoFe (PDB 3U7Q) and VFe (PDB 5N6Y) structures. In both proteins, this includes the helix that covers the front face of the cofactor, most closely by a significant valine side chain near S2B, as marked.

**Figure** **14**, a comparison of the protein structures enclosing the aggregates of yellow waters in the MoFe and VFe proteins, reveals similarities and differences. In both proteins, part of the enclosure includes the helix that covers the front reaction face of the cofactor. This helix includes the valine residue (Val70 in MoFe and Val57 in VFe) that is known to influence catalytic activity: important S2B is also marked on Figure 14. There are clear differences in the protein enclosure of yellow water. In the MoFe protein, there is a long loop of mainly unstructured polypeptide (residues 63–118 in the A chain) that functions as a container for the group of 12 yellow waters further from the cofactor. In the VFe protein, this space is blocked by a helical section (55–65) of the E chain, even though an extended loop in the A chain is still present. This accounts for the smaller aggregate of yellow waters in VFe proteins and also indicates that the larger yellow water aggregate in the MoFe proteins is an artifact of protein structure, without functional significance. Note also the difference in the upper section of yellow waters. In MoFe, a helical section of the B chain provides the wall to yellow water, while in VFe, the wall is a helical section of the A chain. Yellow waters cannot link to the protein surface.

Comparison of Figures 12 and 13 shows clearly a difference in position and connection of the blue water sequence near the cofactor. In the MoFe proteins, the closest four waters of the blue river connect to O4 and O2 of homocitrate. In the VFe proteins, a water in the blue chain is hydrogen bonded to N*ε* of the histidine that ligates V via N*δ*, as well as one yellow water. In the MoFe proteins, the blue water chain is hydrogen bonded to O4 and O2 of homocitrate.

The orange water collections and the proton transfer mechanisms are different in the V and Mo proteins and are detailed in the next section.

## 8 Water Pathways for Proton Transfer

As described in Section 6, the orange and blue rivers radiate in opposite directions away from the cofactor, consistent with the separated pathways for the proton transfer to the catalytic site and the departure of product ammonia. The orange water pathway to the cofactor is now examined and interpreted. The Mo and V proteins are different and are described separately.

### 8.1 Mo Nitrogenase Proton Wire

**Figure 15:**
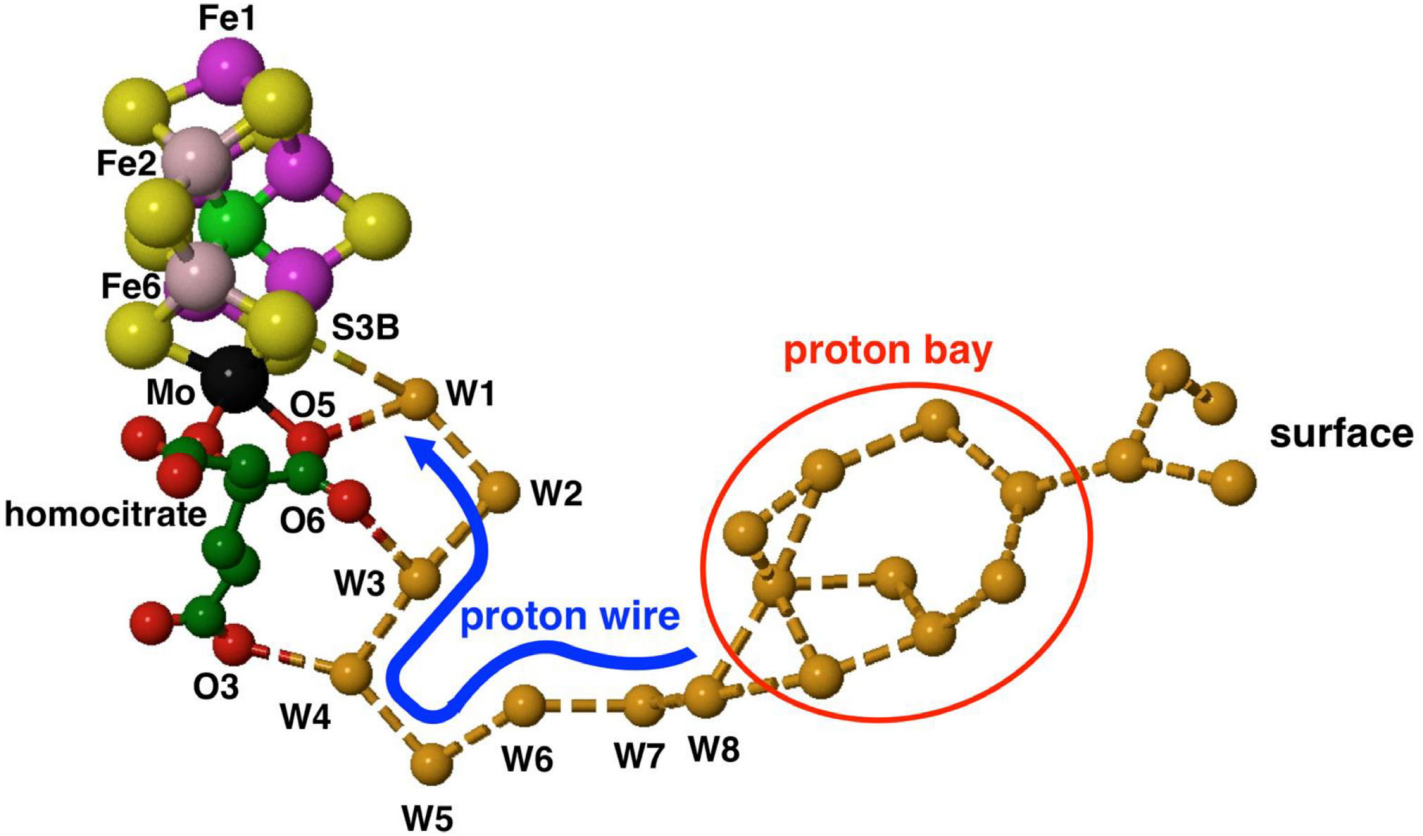
Interpretation of the orange water chain, with a proton bay inside the surface, followed by the proton wire from W8 to S3B.^[^ ^23^ ^]^

In all MoFe proteins, the inner chain of eight waters W1 to W8 is strictly conserved and leads to S3B. The outer section of the orange chain, from W8 to the protein surface, is variable in structure. The outer section contains branches, forms cyclic connections, has a gap in one structure (4WES subunit *γ* ), and harbors exogenous hydrophilic inclusions in two structures. These properties and the variability of the outer section are evident in Figure 11. The presence of hydrophilic inclusions reveals that this outer section of the path is accessible to the external medium. These properties of the outer section, together with the occurrence of bordering aspartate and glutamate sidechains, are consistent with their hosting one or more “protons‐in‐waiting,” ready for controlled transfer from W8 to W1 to S3B. The variable outer collection of orange waters is interpreted as a “proton bay,” supplying the conserved inner single chain as a proton wire to S3B (**Figure** **15**).^[^ ^21^ ^,^ ^23^ ^]^ The species held in the proton bay could be any of the H_2n+1_O_n_^+^ ions (Zundel/Eigen) usually considered for fluid water.^[^ ^15^ ^]^

**Figure 16:**
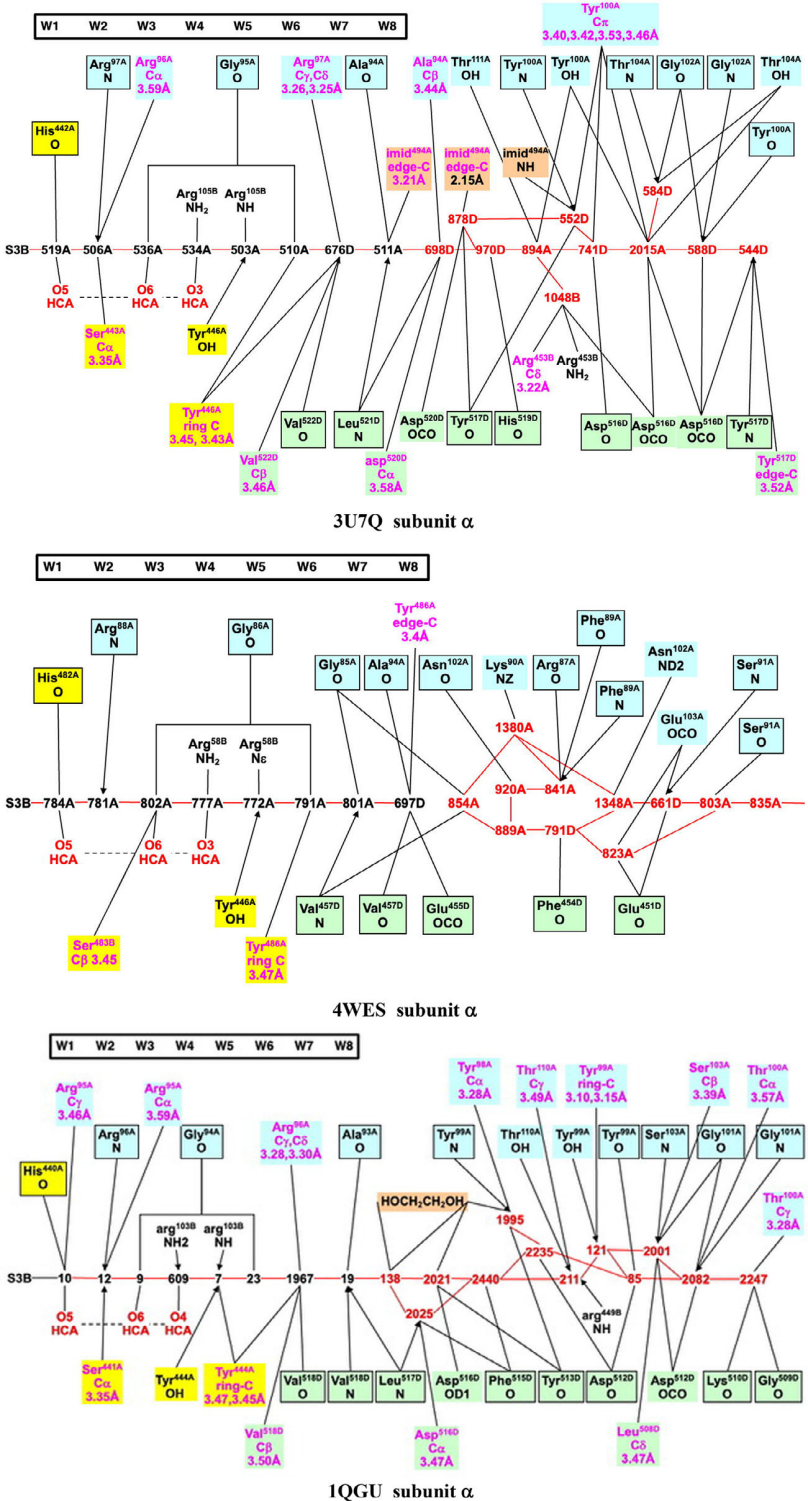
Maps of the hydrogen bonds which define and maintain the orange water chain in MoFe proteins *Av*1 (3U7Q), *Cp*1 (4WES), and *Kp*1 (1QGU). S3B of FeMo‐co is left, protein surface right. Conserved waters W1 to W8 are labeled in black: other waters in the outer section have red labels. Connecting lines are possible hydrogen bonds (<3.2 Å). Arrows indicate probable D–H → A orientations of some hydrogen bonds. Amino acids are colored blue or green to differentiate the chains in which they occur, and those colored yellow are in a short segment near the histidine that ligates Mo. Boxed amino acids use main chain O or NH in hydrogen bonding with water: others use sidechains. Residues engaging possible C–H → O and O → *π* hydrogen bonds are labeled in magenta with distance information: ring‐C refers to the aromatic CH groups of the tyrosine side‐chain, and C*π* to the tyrosine ring face: the distances are C–O as marked. Exogenous inclusions are colored tangerine.

Details of the orange water path and its hydrogen bonding connections with surrounding amino acids in the different MoFe proteins can be presented clearly as connectivity maps. These are shown for one subunit each of the *Av*1, *Cp*1, and *Kp*1 proteins in **Figure** **16**, with possible hydrogen bonds drawn as connecting lines. The conservation of the W1 to W8 sequence of waters (black labels) and of their hydrogen bonding with surrounds is evident on the left side of these maps, and the branching and variability of waters (red labels) in the outer section is also obvious. The protein surface is at the far right in these maps. A small number of surrounding residues may engage C–H → O or O → *π* hydrogen bonds with water molecules in this collection, and these are included (in magenta), with distance information. The connections of the hydrophilic exogenous inclusions ethanediol and imidazolium cation are shown with tangerine coloration. Additional maps presented in ref. [21] demonstrate which three water molecules in the outer section of the *Av*1 protein are displaced in order to accommodate the imidazolium cation and how two water molecules in the outer section of the *Kp*1 protein are substituted by ethanediol. Note that the inclusions are contiguous with W8.

With the large number of possible hydrogen bonds marked in these maps, there are many arrangements of the protons. The directionality of some hydrogen bonds is marked with D → A arrows on these maps. These are main chain N‐H → O, sidechain NH → O, or tyrosine‐OH → O. Protons in the inner W8 to W1 section of the water path have defined positions as they translocate along the proton wire, by the Grotthuss mechanism detailed below in Section 8.2. Contrasting this, in the outer section of the water path, multiple pathways can be constructed for proton movement inwards from the protein surface. There are also multiple opportunities for H_2n + 1_O_n_^+^ entities in the “proton bay.”

**Figure 17:**
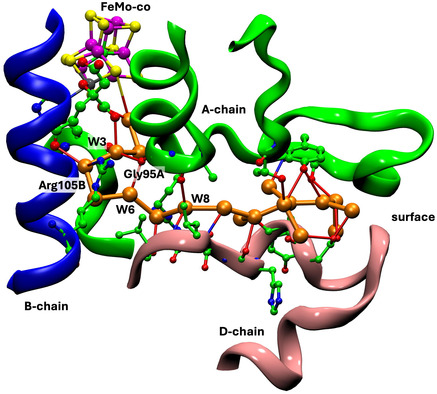
Protein surrounding the orange water chain in PDB 3U7Q subunit *α*.

The structure of the protein surrounding the orange water chain is shown in **Figure** **17** for the *α* subunit of Av MoFe protein (3U7Q). The section from W7 to the protein surface is flanked on one side by unstructured A chain and on the other by unstructured D chain. The inner section of the water chain is different. From W3 to W6, the water chain makes a U‐turn, which is supported and maintained by two significant residues Gly95A and Arg105B. The carbonyl O of Gly95A accepts hydrogen bonds from W3 and W6, while the sidechain of Arg105B donates hydrogen bonds to W4 and W5. Arg105B holds W4 and W5 in place, while Gly95A brings W3 and W6 together, effecting the U‐turn. It is therefore significant that Arg105B is positioned in the long helix of chain B and that Gly95A is located at the end of a helical section of the A chain. This tightness of protein structure around the sections of water chain closer to the cofactor and homocitrate, and looseness of protein structure around the water chain as it approaches the surface is one of the foundations for interpretation of the function of the chain, developed in detail in Section 8.2

**Figure 18:**
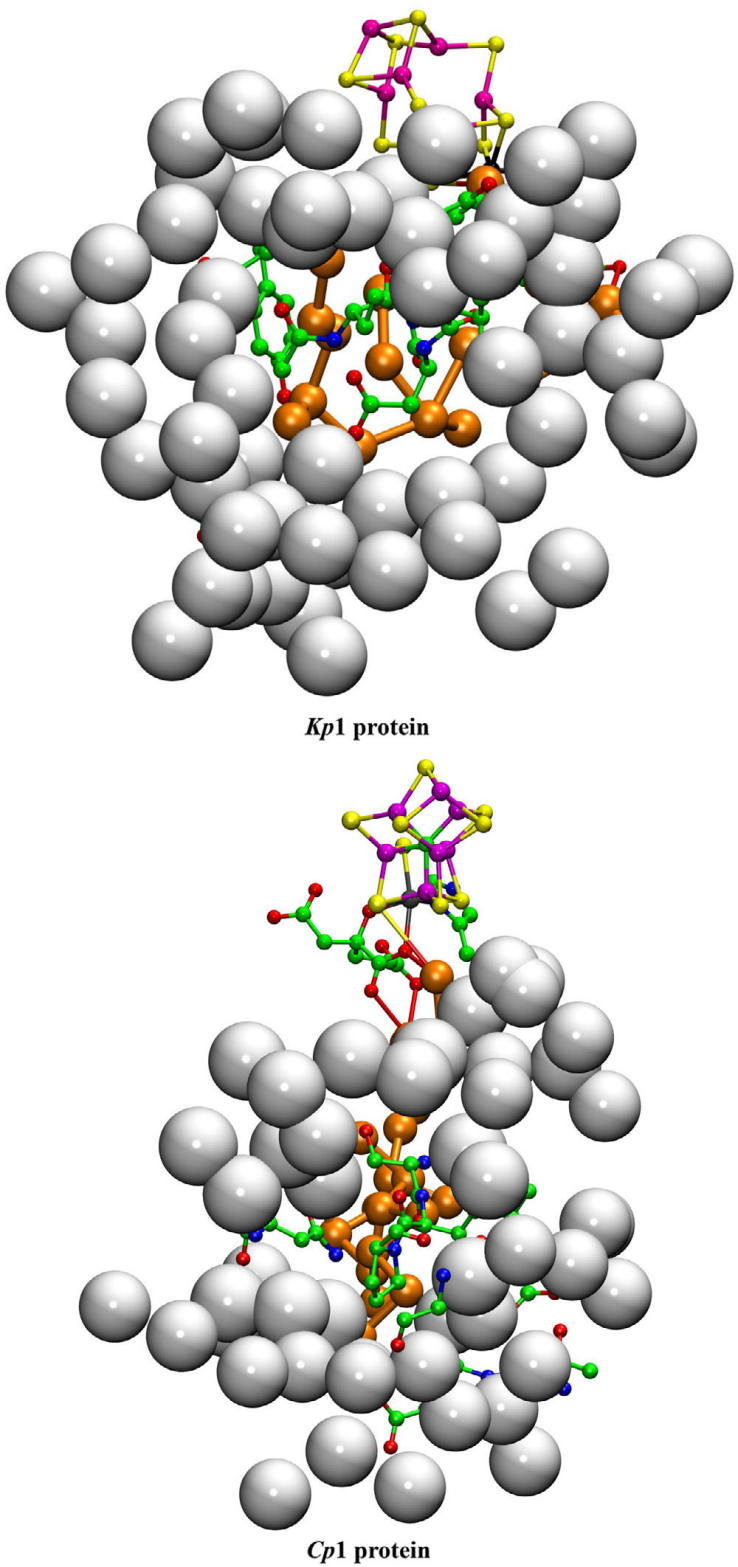
Surface water molecules (white spheres) at the end of the orange water chain in *Kp*1 (PDB 1QGU subunit *α*) and *Cp*1 (PDB 4WES subunit *α*) proteins. The view direction is along the outer section of the water path. Amino acids that surround the water chain at the opening in the surface water sheath are shown: they are Gly509D, Lys510D, Thr511D, Asp512D, and Tyr99A in *Kp*1 and Ser91A, Lys92A, Pro93A, Gly96A, Asn100A, Thr450D, Glu451D, and Glu452D in *Cp*1.

Interesting water arrangements occur where the outer section of this water path reaches the surface of the protein. **Figure** **18** shows the appearance of the end of the orange water path through an opening in the sheath of water molecules (white) on the surface of the *Kp*1 and *Cp*1 proteins. All nearby surface water molecules are included: these openings are definite and uncovered externally. The junction of the water chain and the gap in surface waters is also occupied by hydrophilic and hydrophobic amino acids, marked on Figure 18. Interpretation of this water feature at the mouth of the proton transfer water path is unclear.

### 8.2 Grotthuss Proton Translocation Mechanism, W8 to W1

**Figure 19:**
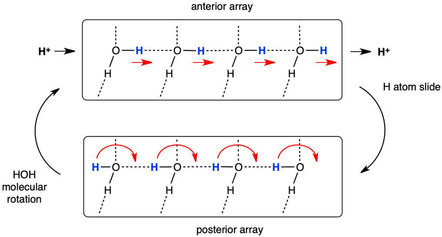
The Grotthuss two‐step controlled relay of protons from left to right along a chain of water molecules. H atom slide converts an anterior array of water molecules to posterior, while HOH molecular rotation converts a posterior array to anterior.

Translocation of protons from the proton bay, along the proton wire W8 to W1 and then to S3B of FeMo‐co, is proposed to occur by the Grotthuss mechanism.^[^ ^14^ ^,^ ^36^, ^37^ ^–^ ^38^ ^]^ The two stages of a Grotthuss mechanism, proton hopping along hydrogen bonds and water molecule reorientation, are shown in **Figure** **19**.^[^ ^23^ ^]^ The anterior array of water molecules can absorb a proton at the entry (left) and release a proton at the exit (right) simply by sliding H atoms along each O–H…O hydrogen bond in the chain. This converts the anterior array to a posterior array. A posterior array is converted back to an anterior array through rotation of each water molecule around the H–O bond that is not in the chain. This rotation, through an approximately tetrahedral angle, transitions through a state in which H is not hydrogen bonded: in contrast, sliding H is hydrogen bonded throughout as O—H—O. This description assumes that each water molecule in the chain accepts one hydrogen bond from the surroundings and donates one hydrogen bond to a neighboring atom, as marked with the extra‐chain connections in Figure 19. Some of these extra‐chain hydrogen bonds can be absent and not affect the mechanism. However, if a water molecule in the chain is involved in two donor or two acceptor hydrogen bonds with the surrounds, the mechanism is disrupted. This is because when a water molecule accepts hydrogen bonds from two external donors both of its H atoms are forced to be in the chain, preventing rotation. And, when a water in the chain forms two donor hydrogen bonds to extra‐chain acceptors, the Grotthuss slide component is unavailable.

**Figure 20:**
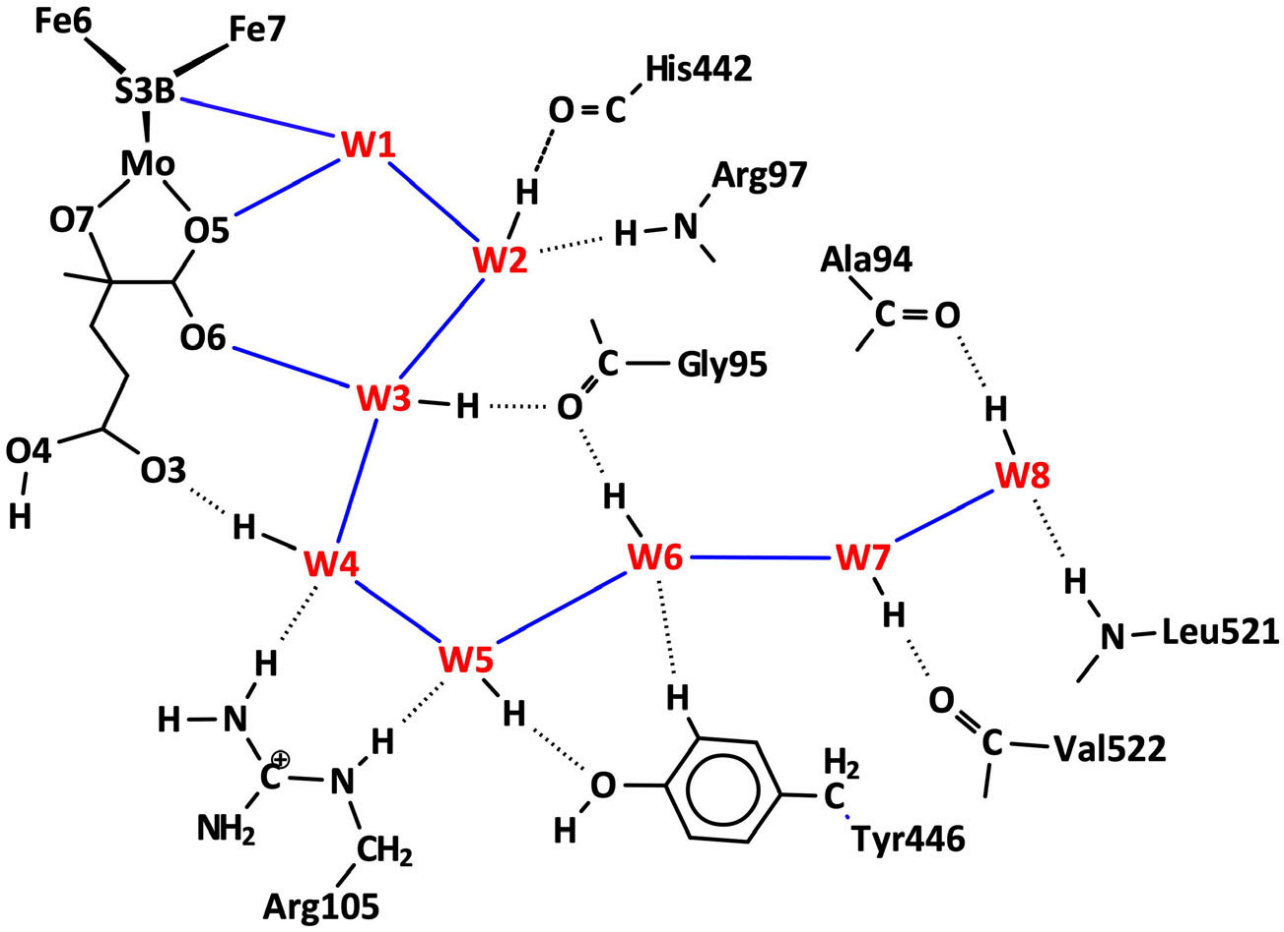
The W1 to W8 water chain and its surrounds in the MoFe proteins, with *Av*1 residue numbering. All waters except W1 form one donor hydrogen bond. Blue connections define the locations of proton slides during the Grotthuss controlled relay of protons.

**Figure** **20** shows how these requirements occur in the framework of the W1 to W8 chain in the MoFe proteins. All waters except W1 form one donor hydrogen bond with a surrounding residue or O3 of homocitrate. Note the special role of Gly95 in accepting hydrogen bonds from W3 and W6, supporting the pronounced bend in the water chain, while Arg105 supports the bend with N‐H donor hydrogen bonds to W4 and W5. The blue connectors in Figure 20 define the locations of proton slides. Note that sliding also occurs between W3 and O6 of homocitrate: this is reversible sliding, with O6 functioning as a temporary site for a proton during the complete mechanism. A complete description of hydrogen bonding distances and angles at each water in the W1 ‐‐ W8 chain is provided in ref. [23]

Detailed analysis of the operation of the Grotthuss mechanism in the *Av*1 MoFe protein has been published.^[^ ^23^ ^]^ Density functional simulations of proton translocation used a large 269‐atom model that included all residues hydrogen bonded to and surrounding the water chain. A specific 20‐step mechanism was developed to take H_3_O^+^ at W8, through W7 to W1, and finally to H bonded to S3B. Some key results from this analysis, pertinent in the present context, are summarized. (1) HOH rotations involve extensions of up to 3.6 Å between contiguous water molecules. (2) H atom slides pass through short (ca. 2.5 Å) O–H–O hydrogen bonds. (3) HOH rotations and H atom slides have low potential energy barriers, <7 kcal mol^−1^. (4) O6 and O5 of homocitrate are relatively basic sites that can be protonated by H_3_O^+^ occurring at positions W3 and W1 respectively: O6 and O5 provide a buffering facility for proton translocation. (5) H_3_O^+^ is stabilized at the W6, W3, and W1 positions of the water chain, any of which could be a ’resting state’. (6) W1 is a versatile agent for the transfer of protons to atoms of FeMo‐co, because W1 is not required to form a constraining hydrogen bond with any of its neighbors and can easily reorient. (7) The geometry of the space containing W1, W2, W3, O5, and O6 is propitious for H transfer steps. (8) The largest potential energy barrier is 14 kcal mol^−1^, for the final proton slide from the W1 position to S3B. This step this involves a shortening of W1‐‐S3B by ca 1 Å.

**Figure 21:**
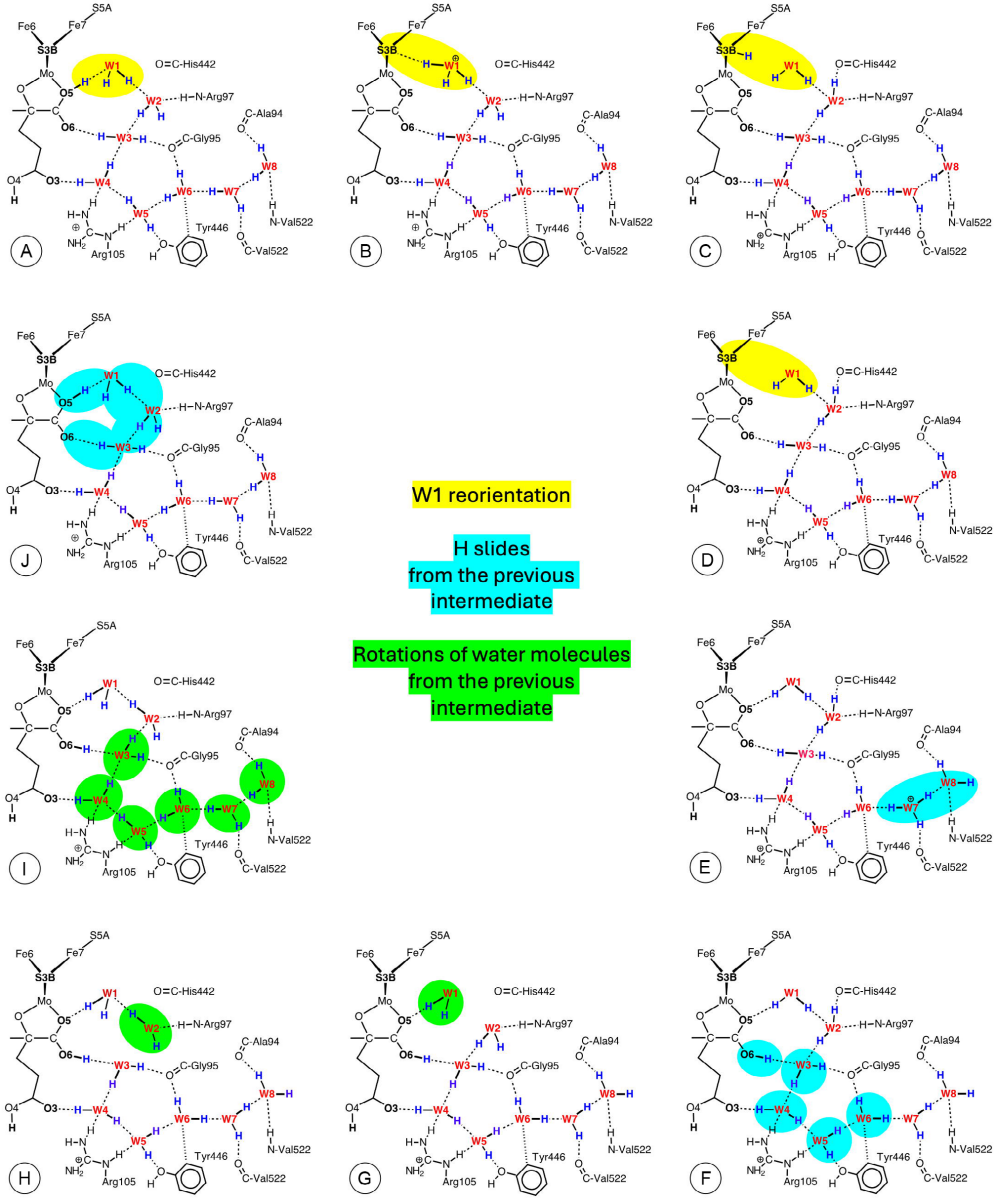
The main intermediates and steps in the calculated Grotthuss mechanism for operation of the proton wire in *Av*1 Mo nitrogenase.^[^ ^23^ ^]^ Yellow background highlights steps that occur at W1 and S3B, including reorientation of W1 and H transfer to S3B; cyan backgrounds highlight H slide steps that have occurred from the previous intermediate; green backgrounds highlight rotations of water molecules that have occurred from the previous intermediate. A) is the relatively stable resting state, with protonated O5, which protonates W1 B), which transfers to S3B C). After reconfiguration of S3B‐H and migration of the H atom away from S3B D), proton addition at the W8 end of the chain causes spontaneous proton slide to W7 E). A sequence of proton slides leads to protonation of O6 F), at which point all waters are primed for rotation steps, the first of which occurs at W1 G). Then, W2 rotates H), followed by rotations at W3, W4, W5, W6, W7, and W8 I). A concerted sequence of proton slides, from O6 to W3 to W2 to W1 to O5 generates J), which is the same as (A).

Steps in the Grotthuss mechanism for the proton wire of *Av*1 Mo nitrogenase are outlined in **Figure** **21**. The steps are color differentiated as W1 reorientations—yellow, proton slides—cyan, and HOH rotations—green. An outline of the sequence through intermediates **A** to **J** is contained in the caption to Figure 21.

What drives proton transfer along the proton wire in the Mo enzyme? Overall, the nitrogenase reaction uses electrons and protons, and it is usually assumed that the steps are proton coupled electron transfers, according to the PCET paradigm.^[^ ^39^ ^]^ I have examined this question and shown that there is a trigger for the last stage of the transfer, from W1 to S3B of the cofactor.^[^ ^40^ ^]^ Calculations of cluster charge distribution upon electron addition reveal that the added negative charge is on the S atoms of FeMo‐co, which thereby become more basic and able to trigger proton transfer from H_3_O^+^ waiting at the near end of the proton wire. This mechanism is supported by calculations of the dynamics of the proton transfer step, in which the barrier is reduced by ca 3.5 kcal mol^−^^1^, and the product stabilized by ca 7 kcal mol^−^^1^ upon electron addition. Also, H atom quantum tunneling is probable in this final W1 to S3B step but less probable at other places along the proton wire because substantial movements of the heavier O atoms are involved.

### 8.3 V Nitrogenase

**Figure 22:**
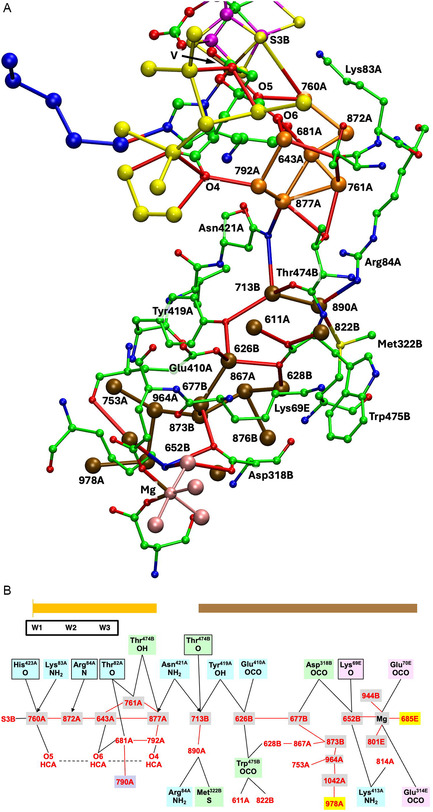
A) The orange and brown collection of waters near FeV‐co in the *α* subunit of the *Av*1 VFe protein (PDB 5N6Y), with possible hydrogen bonding connections (<3.2 Å). B) Map of the hydrogen bonding connections in A. Amino acids are color coded by chain, with main chain atoms boxed. Waters shaded yellow are at the surface of the protein, and waters that can be construed as mainly on a path from S3B on the left to these surface waters are shaded gray. Water 790A, hydrogen bonded to 681A, links the orange waters to the yellow water collection. The orange stripe and labels W1, W2, and W3 mark the similarities with the orange water path of the MoFe proteins.

The water structure and the options for proton transfer to FeV‐co in V nitrogenase are markedly different from those just described for Mo nitrogenase. In Section 7.2, the yellow water aggregates around HCA in the VFe proteins are shown to be compact and to not extend to the protein surface. Therefore, yellow water is not likely to be part of the mechanism for serial transfer of external protons to FeV‐co. What about the orange waters, which have W1, W2, and W3 in common with the Mo proteins? Figure 9 above demonstrates the different directions of the orange rivers in V and Mo proteins. The more significant difference is that the water collection in the VFe protein does not have the continuous sequence of the MoFe proteins. **Figure** **22**A shows details of the water structure in the *α* subunit of the *Av*1 VFe protein (PDB 5N6Y): the *δ* subunit is very similar. There are seven connected waters (orange) in the vicinity of FeV‐co and homocitrate, then a gap without water–water hydrogen bonds to the pair 713B and 890A (brown), and then another gap to a large collection also colored brown. At the lower end of this collection resides exogenous Mg^2^^+^ coordinated by four water molecules (pink). A connectivity map (Figure 22B) is provided as an aid to understanding the full structure here.

This water structure is analyzed and described with an expectation that it will reveal a feasible pathway for proton transfer from protein surface to the cofactor FeV‐co. The first three waters, 760A, 872A, and 643A, are very similar in position and connections to W1, W2, and W3 in the MoFe proteins. They could similarly function as the final agents for proton transfer to S3B in FeV‐co. However, the next four orange waters (761A, 877A, 792A, and 681A) are different, branching the chain and forming different hydrogen bonding connections to O6 and O4 of homocitrate. Also, water 681A in this set is within hydrogen bonding distance of 790A in the yellow water aggregate. Direct orange‐yellow hydrogen bonding does not occur in the MoFe proteins.

Looking beyond the orange collection in Figure 22, there is one significant linkage between the orange waters and the first pair of brown waters, being the bridge between waters 877A and 713B formed by two N‐H bonds of the NH_2_ sidechain of Asn421A. In addition, Thr474B can also form hydrogen bonds with 877A and 713B, although this linkage uses the mutually separated main chain CO and sidechain OH functions. Following 713B, there is another gap along the water chain (shaded gray in Figure 22B), bridged by the sidechain OH of Tyr419A between waters 713B and 626B. Another gap in direct water–water hydrogen bonding, from 677B to 652B, is bridged by the two sidechain carboxylate O atoms of Asp318B. At this point, the brown waters are connected to the hydration sphere of Mg^2^^+^ at the protein surface. A chain of waters branching at 677B leads through 873B, 964A, and 1042A to water 978A which is also on the surface of the protein but not near Mg. The two water molecules at the protein surface are emphasized with yellow shading in Figure 22B.

### 8.4 An Alternative Grotthuss Proton Translocation Mechanism for the V Enzyme

I now evaluate the possibilities for proton transfer from the protein surface to S3B of FeV‐co, guided by the Grotthuss mechanism already described for the MoFe proteins. Water molecules on the likely pathway are shaded gray in Figure 22B. A continuous sequence of hydrogen bonded water molecules does not occur: there are two disruptions, one bridged by NH_2_ of the amide sidechain of Asn421A and the other by OH in the sidechain of Tyr419A. Can these bridges transfer protons and integrate with Grotthuss steps?

**Figure 23:**
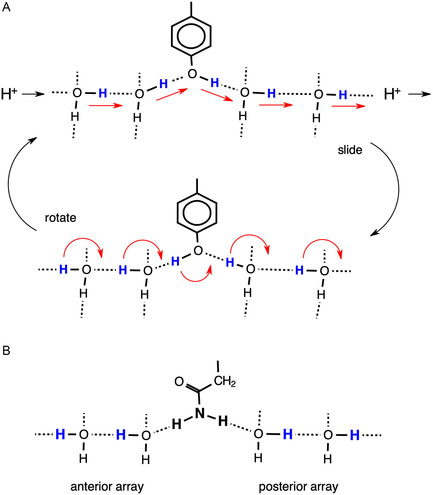
A) The OH function of a tyrosine sidechain bridging a water chain can participate in Grotthuss slide and rotation operations. B) The NH_2_ function of an asparagine sidechain bridging a water chain blocks the Grotthuss slide and rotation operations.

The Tyr sidechain bridge is able to sustain the Grotthuss slide and rotation operations, as illustrated in **Figure** **23**A. However, the NH_2_ function of the Asn sidechain forms donor N‐H‐‐O hydrogen bonds to *both* adjacent waters (Figure 23B) and thereby blocks both the slide and rotate operations of the Grotthuss mechanism. Referring to Figure 19, this bridge enforces simultaneous anterior and posterior orientations on the adjacent waters, preventing their alternation and negating the Grotthuss mechanism. This is analogous to the role of asparagine in directing the hydrogen bonding pattern in the aquaporin water channel in order to prevent proton translocation.^[^ ^41^ ^]^ Might the bridge reconfigure or twist to allow a different type proton transfer to occur? This is very unlikely because the geometry of the H_2_O‐HNH‐OH_2_ bridge is tight at both occurrences in the 5N6Y protein, with perfect planar stereochemistry at N, O‐‐N distances 2.88−2.98 Å and C–N‐O angles 114–123°. Also, the very large pKa of an amide (>16) discounts Asn N‐H → OH_2_ transfer.

**Figure 24:**
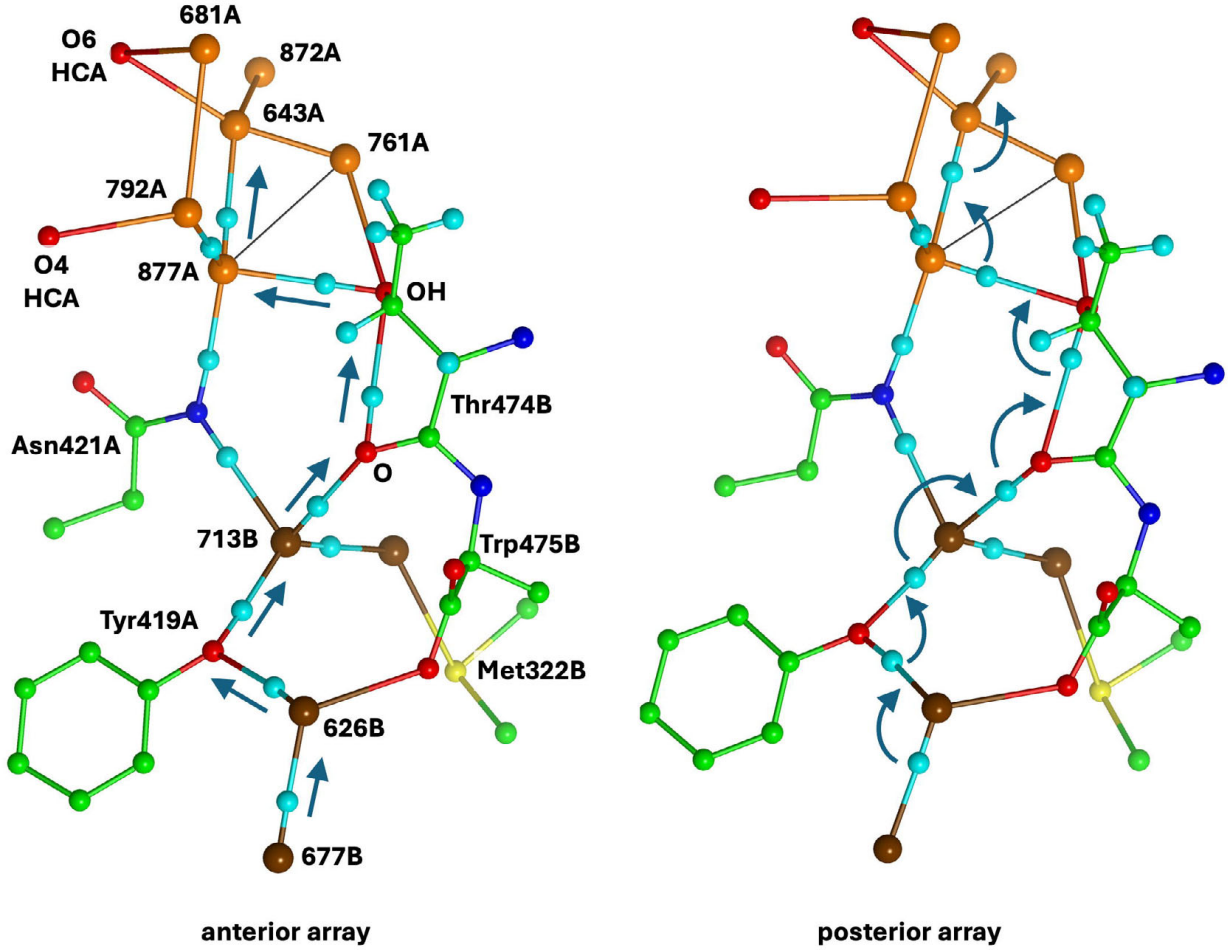
Part of the crystal structure of VFe protein 5N6Y, with key H atoms (cyan) added along hydrogen bonds. Water molecules are colored orange and brown as in Figure 22. The labeled main chain carbonyl O and the side chain OH of Thr474B are key components. The left image shows H atoms at the anterior location of each water or other hydrogen bonding site in the sequence from 677B to 643A, with arrows marking the Grotthuss slides. The right image shows the Grotthuss rotations that follow these slides.

Here is an alternative Grotthuss mechanism, using Thr474B instead of Asn421A to link water 713B and water 877A. This mechanism is described in **Figure** **24**, which shows an extract of the 5N6Y crystal structure (*α* subunit) with H atoms added to relevant sections. The orientation of the two images in Figure 24 is similar to that of Figure 22. The carbonyl O of Thr474B is 2.72 Å from water 713B, the sidechain OH of Thr474B is 2.99 Å from water 877A, the carbonyl O and OH are separated by 2.99 Å, and the interatomic angles in this set of four O atoms are 95 and 93°. Clearly, the set of four atoms—713B, Thr474B‐O, Thr474B‐OH, and 877A—can support a sequence of hydrogen bonds, 713B to carbonyl O to sidechain OH to 877B. The left image in Figure 24 shows the anterior array of seven H atoms in sequence from water 677B to water 643A, with arrows on the slides they can undergo. This generates an H atom array in posterior positions, shown in the right image. Each of these can then rotate through an approximately tetrahedral angle, as arrowed on Figure 24‐posterior, generating an anterior array and delivering one H to beyond water 643A. This is Grotthuss slide and rotate. Two OH functions (Tyr419A and Thr474B) are involved, and the one O–C bond of each is the analog of the hydrogen bond accepted by each water molecule in a standard water chain (Figure 19). This mechanism is nonstandard because it involves a main chain carbonyl O atom as a transfer agent, with incorporation of an inserted proton such that this C=O operates as C=OH. The low basicity of C=O suggests that this mechanism is less efficient than the standard Grotthuss mechanism of the continuous water path in the MoFe proteins.

This mechanism provides an answer to the question about the sources and pathways for serial transfer of the multiple protons required in the catalytic reactions, physiological and nonphysiological, of V nitrogenase. This mechanism postulates the pathway for proton transfer from the brown water collection near the protein surface to the orange water set close to the vanadium cofactor. This FeV‐co orange collection differs from the single chain in the MoFe proteins, and several options are conceivable for the final stage of proton transfer to FeV‐co. However, water 643A is the equivalent of W3 in the MoFe proteins, and the final transfer from W3 to W1 and then to S3B is probably the same in the two isozymes.

## 9 Water Pathways for NH_3_ Egress

I now elaborate the structure and function of the blue river that is connected to the cofactor on the opposite side to the proton wire orange river. As intimated, the proposed function is facilitation of the removal of NH_3_ produced in the Fe2‐Fe6 reaction zone. Water in the blue river does not “dissolve” NH_3_, nor does it move out of its “gorge” to allow NH_3_ to pass. Water molecules in the blue river, in conjunction with main chain and sidechain atoms of the surrounding amino acids, provide hydrogen bonding pivot points, and the NH_3_ molecules swing their hydrogen bonds from one pivot point to another along the river and its banks. This choreography for translocation of NH_3_ molecules does not require that the river be a continuous sequence water molecules with intra‐river hydrogen bonds, and breaks (“waterfalls”) can and do occur. The egress of NH_3_ has been studied in depth, and for the *Av* MoFe protein, a detailed mechanism has been reported.^[^ ^22^ ^]^ Before describing this mechanism, it is appropriate to examine and compare the structures of the blue rivers as they occur in the Av, Cp, Kp MoFe, and Av VFe proteins.

### 9.1 Blue River Details and Variations

**Figure 25:**
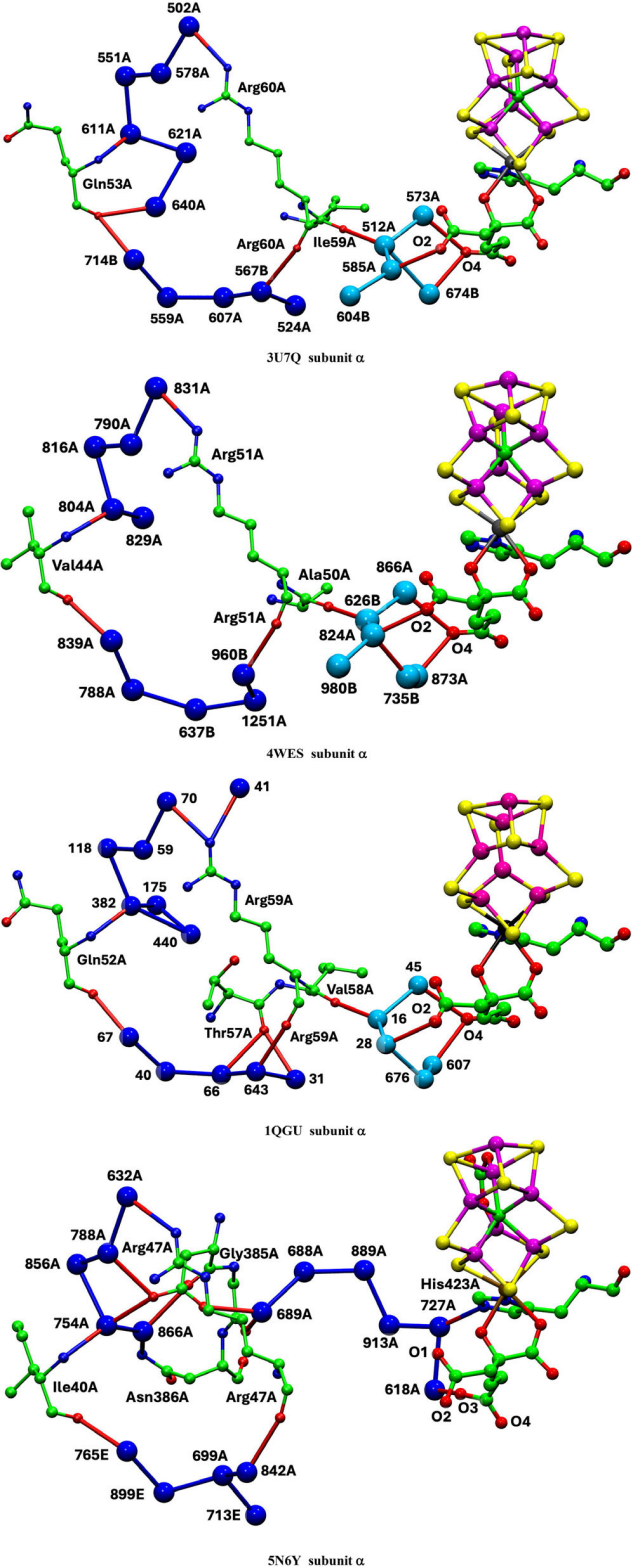
The blue rivers in the MoFe *Av*, *Cp*, and *Kp* proteins and the *Av* VFe protein, with water molecules labeled. To aid comparison, all views have the same orientation relative to the cofactor. In the three MoFe proteins, the lighter blue spheres are water molecules hydrogen bonded to homocitrate and linked to the darker blue chain via the bridging residues marked. The VFe protein differs, with the proximal component hydrogen bonded to N*ε* of His423A.

**Figure** **25** shows the blue river as it occurs in the *α* subunit of the four crystal structures 3U7Q, 4WES, 1QGU, and 5N6Y. To aid comparison, all are viewed with the same orientation relative to the cofactor. In the MoFe proteins, there is a set of five or six water molecules hydrogen bonded with the O4 and O2 atoms of homocitrate and not directly hydrogen bonded to the nearest waters of the blue river. This set is colored lighter blue in Figure 25. As described in Section 6.2 and 6.3, the VFe protein is quite different in this region near the cofactor. The blue river starts in a different space, from N*ε* of the His ligated to V, with only one connection to homocitrate.

In all four proteins, after the first section close to the cofactor, there is an interruption before the next group of four or five waters. In all four this interruption is bridged by O of an arginine (Arg60A, Arg51A, Arg59A, and Arg47A, respectively), and in the MoFe proteins, there is also a bridge by O of a hydrophobic residue (Ile, Ala, and Val). In all proteins, the side chain of the bridging arginine is hydrogen bonded to the last water in the blue river. After the first interruption, there is a second break in the blue river, bridged in all four proteins by the mainchain O and NH of one residue (respectively Gln53A, Val44A, Gln52A, and Ile40A). The last section of the blue river meanders with variations and terminates in an unstructured region near the protein surface.

**Figure 26:**
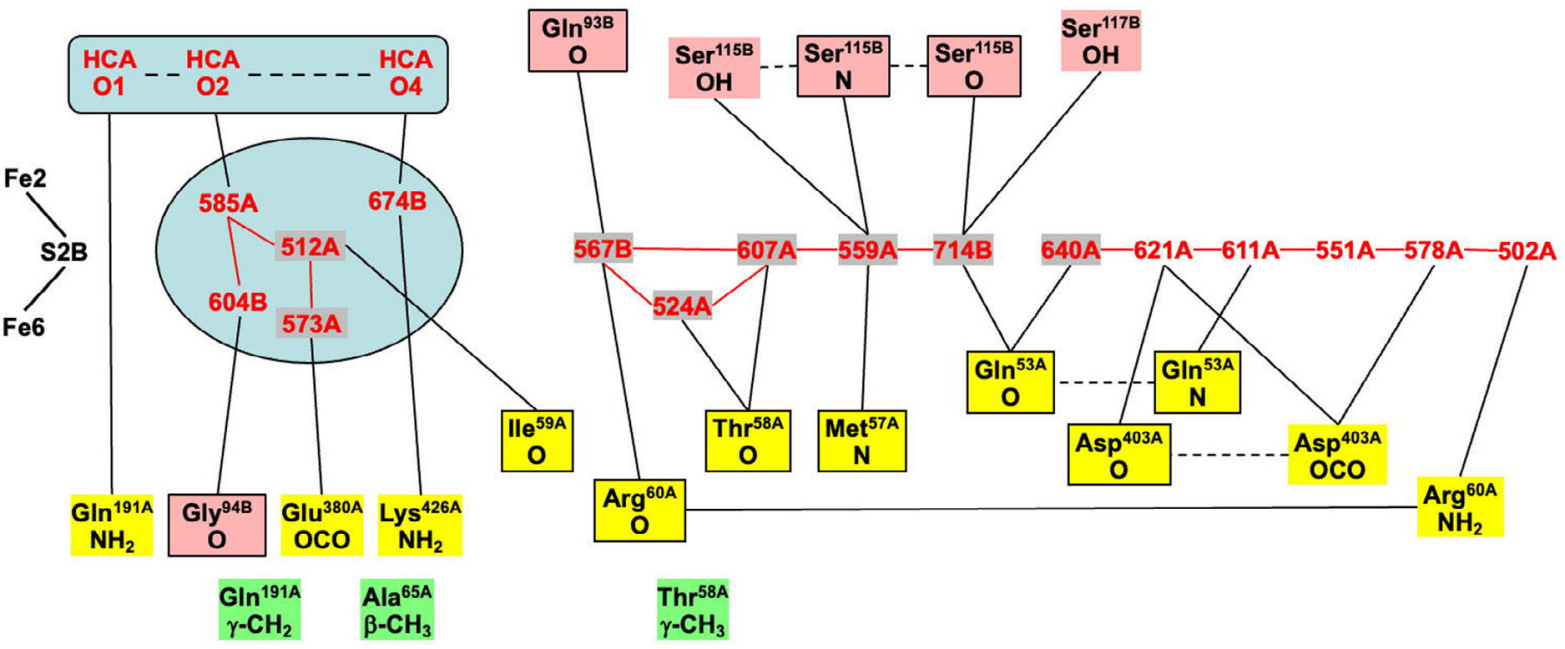
Map of the blue river and its connections in the *α* subunit of crystal 3U7Q. Water molecules are labeled red; protein atoms are differentiated as chain A yellow and chain B salmon, with main chain atoms boxed and side‐chain atoms unboxed; connecting lines are possible hydrogen bonds. The groups colored green are hydrophobic side‐chain components that are potential steric barriers. The aqua enclosure contains the light blue waters in Figure 25.

The complete surroundings and hydrogen bonding of the blue river in the *α* subunit of crystal 3U7Q are mapped in **Figure** **26**. The other MoFe proteins are similar.

**Figure 27:**
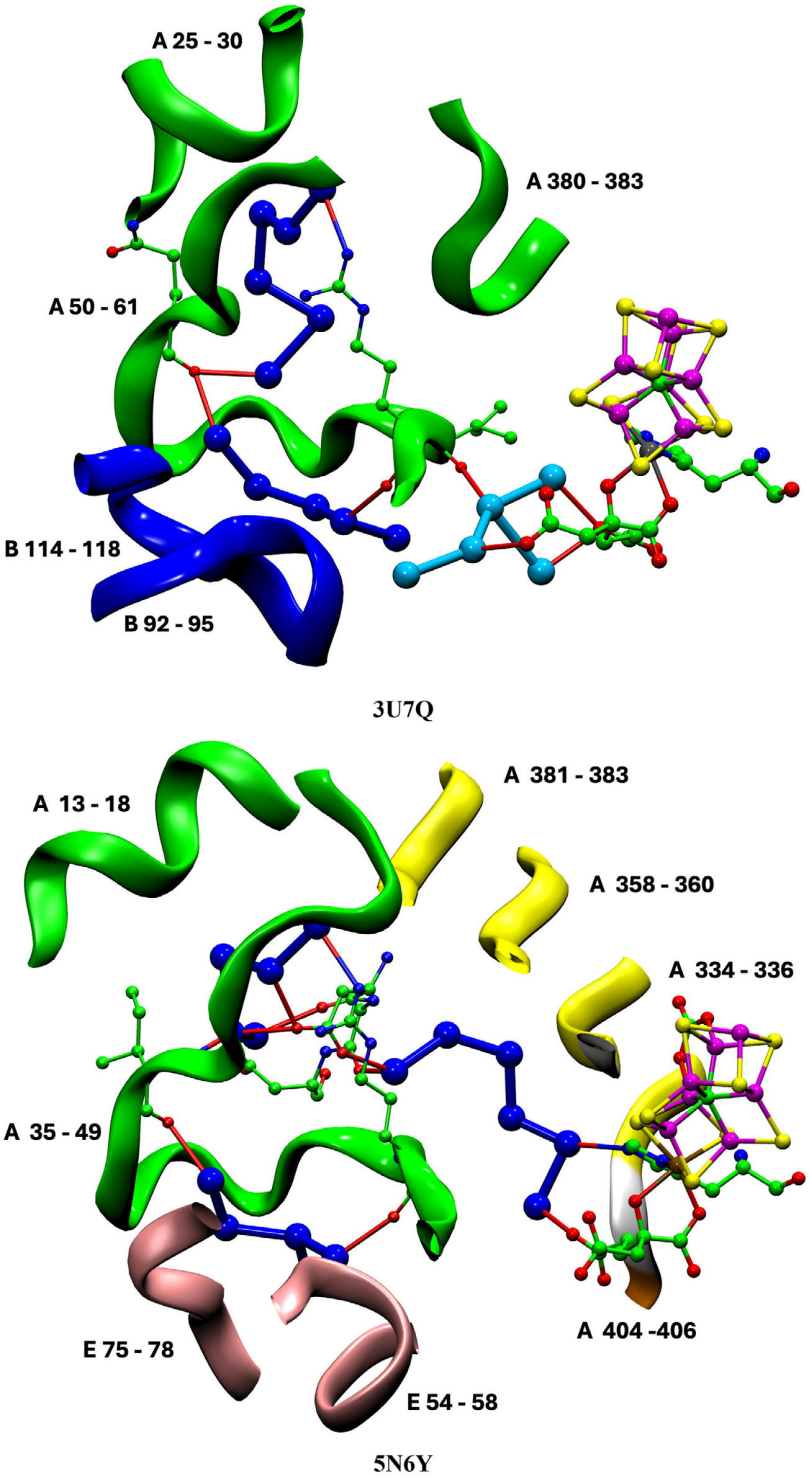
Protein surrounding the blue river in MoFe (PDB 3U7Q) and VFe (PDB 5N6Y). The yellow segments are part of a *β*‐sheet.

**Figure** **27** depicts the sections of polypeptide chain that surround water molecules in the blue rivers of the MoFe and VFe proteins (*α* subunit). There are similarities on the left and lower left sections of these pictures and a clear difference on the right side, where the A chain is unstructured in MoFe but is part of a *β*‐sheet in VFe. The A334−339 component of this sheet envelops the distinctive CO_3_ ligand of the *V* cofactor.

### 9.2 Mechanism of NH_3_ Egress

The previous investigation^[^ ^22^ ^]^ of the channel for egress of product ammonia started with a search for cavities in the *Av* MoFe protein in the vicinity of FeMo‐co and the water around homocitrate. The catalytic cycle of Mo nitrogenase generates NH_3_ in the Fe2 ‐ Fe6 space,^[^ ^26^ ^,^ ^28^ ^]^ and the NH_3_ is released into a hydrophobic anhydrous space, away from the *exo* position of Fe6. Then the initial movement of NH_3_ would be into the rich hydrogen bonding environment of the collection of waters around homocitrate.

**Figure 28:**
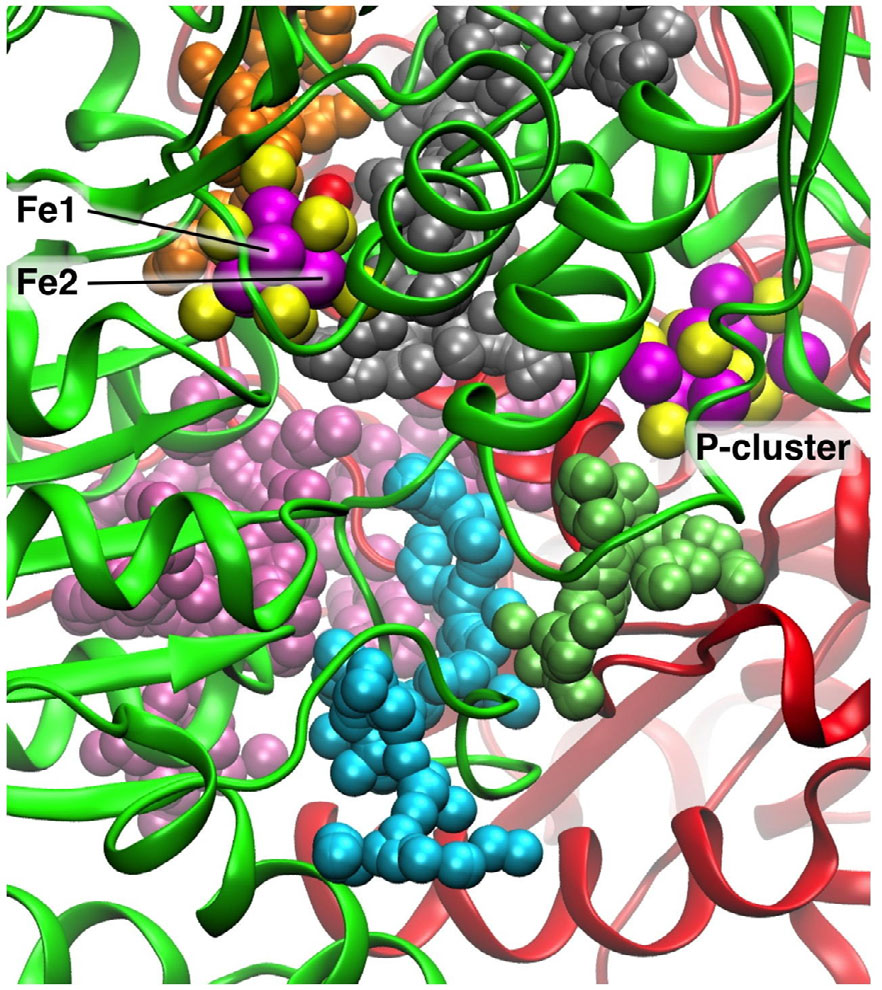
Relevant pockets calculated for *Av* protein, PDB 3U7Q. The pockets are represented as assemblies of alpha spheres, differentiated by color. The view direction is directly along the pseudo‐threefold axis of FeMo‐co, from the top: Mo and homocitrate are on the distant side of FeMo‐co. The rotational orientation of FeMo‐co is signified by the marked position of Fe2 and by the location of the P‐cluster. The silver assembly is the location the homocitrate water pool, and the orange assembly is the proton wire water chain. The two protein chains that envelop the pockets are colored green A) and red B). From ref. [22].

Possible spaces in the protein for the continuing movement of NH_3_ were explored in the following way. The software fpocket^[^ ^42^ ^]^ was used to find alpha spheres, which are empty spheres possessing four atoms equally distant from the sphere center. Using alpha spheres with a minimum radius of 2.8 Å, pockets or channels in the protein were identified, as aggregates of at least 35 intersecting alpha spheres. These are sufficiently large to contain NH_3_ or NH_4_^+^ and possibly contain water. The calculations were made for all high resolution crystal structures of the MoFe proteins available in 2013 and checked for species generality. The following results are for crystal 3U7Q. **Figure** **28** shows the resulting color coded collections of alpha spheres, as viewed along the pseudo‐threefold axis of FeMo‐co, from the top. The silver pocket contains the water molecules of the homocitrate pool, while the orange pocket extending upwards from FeMo‐co in Figure 28 is the water path that supports the proton wire. The mauve pocket and the lime pocket could be discounted because intervening protein blocked them from possible openings to the silver pocket. The key pocket is that colored cyan, which extends from an edge of the silver pocket, twisting towards the surface of the protein. The inner section of the cyan pocket is very similar for the *Av* and *Kp* proteins.^[^ ^22^ ^]^

**Figure 29:**
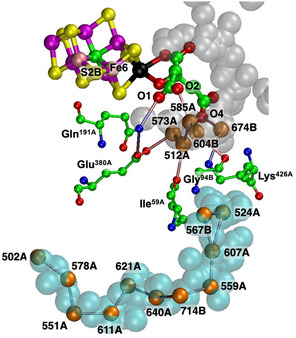
The silver and cyan pockets in *Av* MoFe protein (3U7Q), together with the associated water molecules (all colored orange). The five waters in the silver pocket and linked to O2 and O4 of homocitrate are the light blue set in Figure 25, and the water molecules in the cyan pocket are the dark blue set in Figure 25.

**Figure** **29** shows the water molecules associated with the silver and cyan pockets. These are the waters of the blue river. The five waters that link to O2 and O4 (marked as light blue in Figure 25) are in the silver pocket. The gap between the silver and cyan pockets is surrounded by but not blocked by Lys426A, Ile59A, and Gly94B. Then, across this gap, the sequence of eleven blue river waters, from 567B and 524A and continuing to 502A, follows the cyan pocket. The co‐location of the cyan alpha spheres and the eleven water molecules that are in or beside them is a confirming definition of the passageway for NH_3_.

With this knowledge of the passageway, the positions of NH_3_ and its hydrogen bonds could be modeled. The starting point had NH_3_ just dissociated from Fe6 (Fe6‐‐NH_3_ = 2.7 Å) and NH_3_ hydrogen bonded to both S2B and O1 of HCA. Through this first stage of NH_3_ movement, the important surroundings are O1, O2, and O4 of HCA, the side‐chain CH_3_ of Ala65A, and both the terminal amide group and the CH_2_ functions of the side chain of Gln191A. During this stage, reconfiguration of the side chain amide group of Gln191A would improve hydrogen bonding with passing NH_3_.

NH_3_ was stepped through a sequence of positions, relinquishing previous and forming new hydrogen bonds. A significant property of the passageway is the prevalence in the surrounds of main chain carbonyl functions, as potential hydrogen bond acceptors. In positions through the cyan cavity domain, N‐H → OH_2_ hydrogen bonds are also part of the model. These hydrogen bond acceptors and donors on the surrounding residues can be traced in Figure 26. The ability of water molecules in the chain to accept one such hydrogen bond at each position of NH_3_ is favorable in terms of hydrogen atom accounting, because each water can form two other donor O‐H → O hydrogen bonds, one to the next water in the chain and one to protein main chain carbonyl oxygen. Displacements of water molecules by 0.4—1.0 Å are involved in the model, and it was clear that some local geometric reorganization would be required as NH_3_ is passed along this pathway. Protein fluctuations as part of the mechanism are readily incorporated because different chains with almost no tertiary structure surround the pathway (Figure 31).

**Figure 30:**
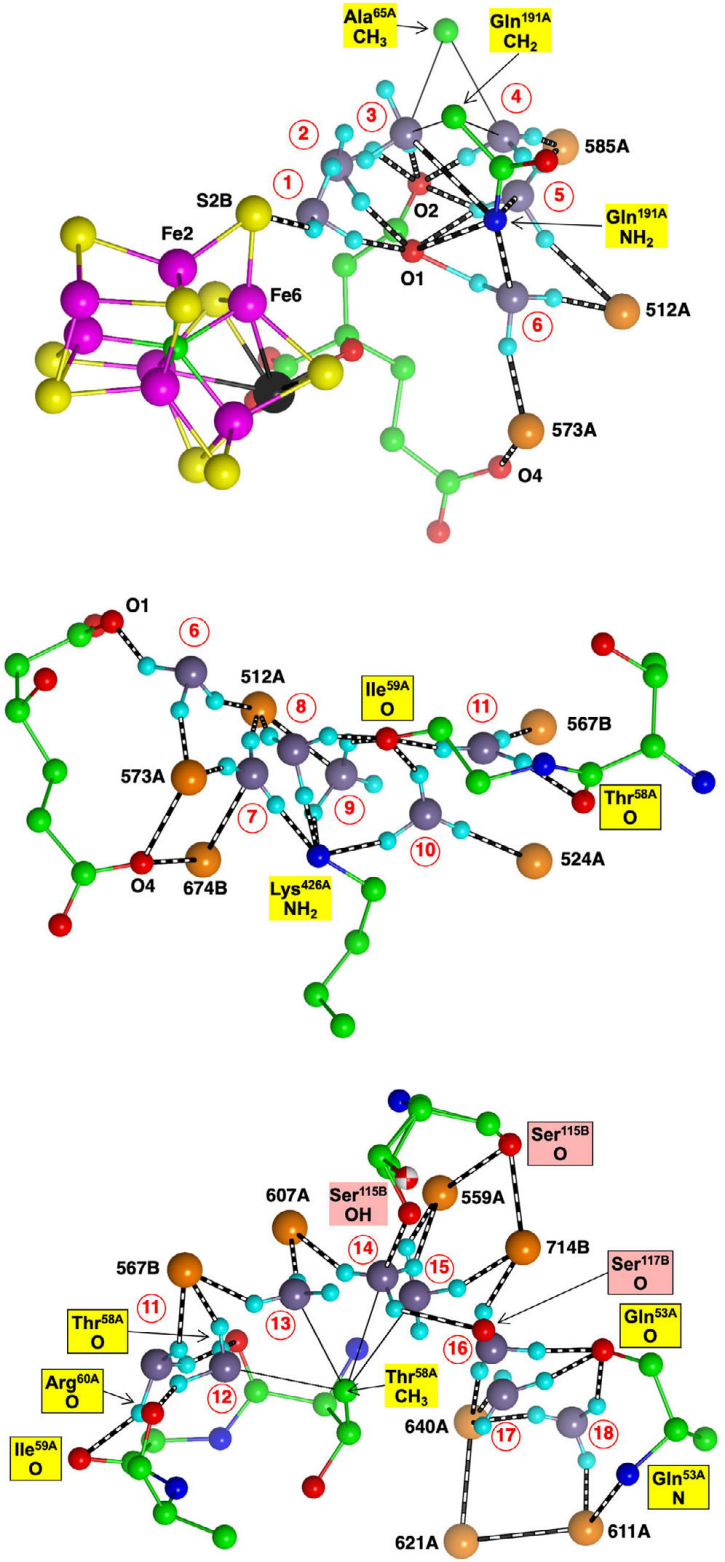
The sequential positions (numbered 1–18) constructed for NH_3_ in the egress pathway. The pictures are simplified in order to maintain the clarity of the multiple positions for NH_3_ and its hydrogen bonds (black and white). H atoms on the water molecules and their hydrogen bonds are not shown, for clarity. The thin lines to *β*‐CH_3_ of Ala65A and *γ*‐CH_2_ of Gln191A signify steric boundaries for NH_3_ at positions 3 and 4. The side chain CH_2_OH function of Ser115B is disordered in PDB 3U7Q, and both configurations are shown (beachball and filled): only one forms a good OH → NH_3_ hydrogen bond at position 14. The lines from side chain CH_3_ of Thr58A are contacts of ca 2.7 Å to N in positions 12, 13, 14, and 15 and are possible C–H → NH_3_ hydrogen bonds.

**Figure 31:**
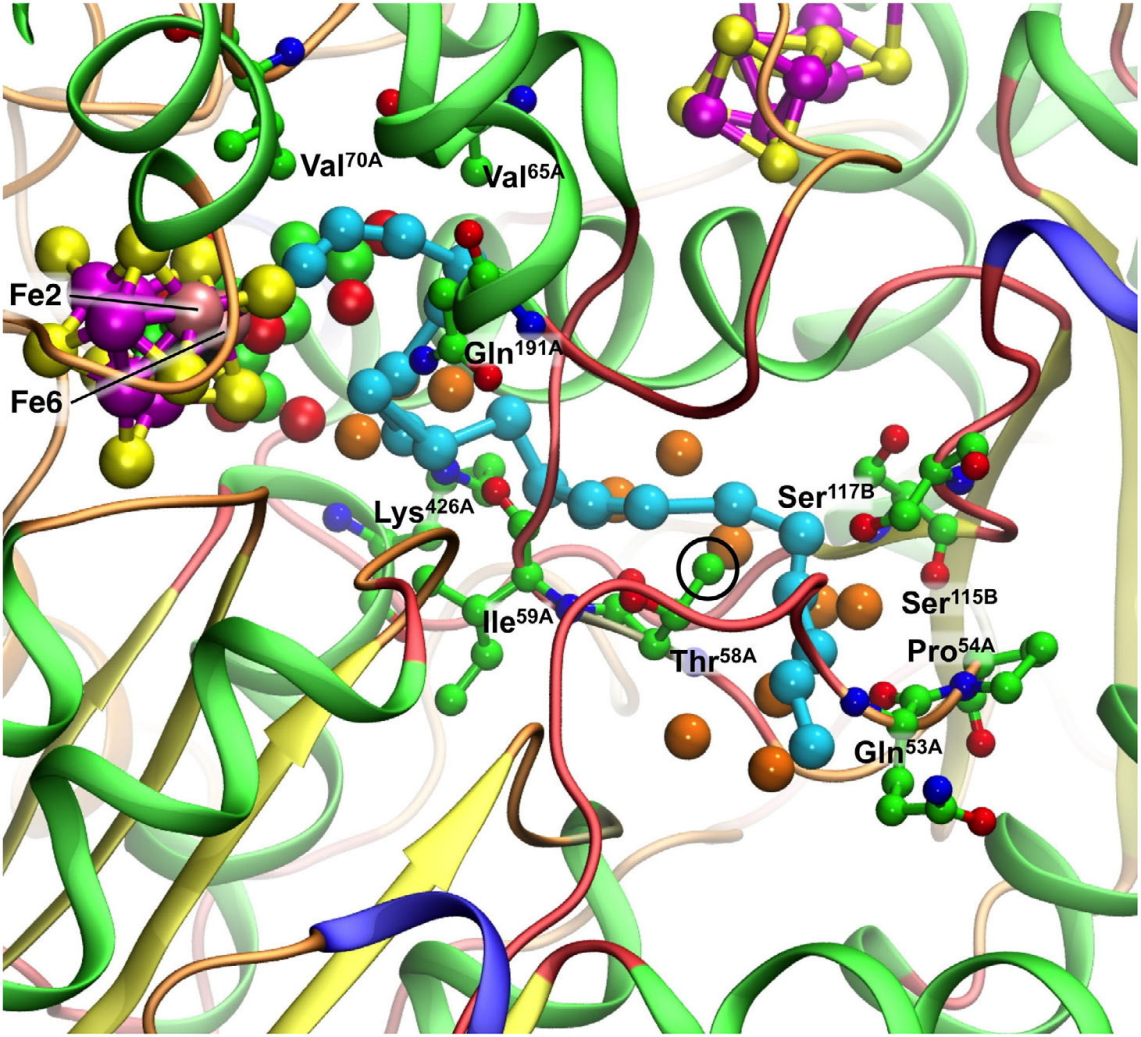
The sequence of modeled positions for NH_3_ from the reaction face of FeMo‐co towards the protein surface. Cyan spheres are the locations of N atoms: H atoms are not shown. Key interacting amino acids are marked and labeled. The side chain CH_3_ of Thr58A (circled) is significant, because the lone pairs of NH_3_ looping around it are 2.6—2.9 Å distant, with the potential for formation of weak C–H → NH_3_ hydrogen bonds. Protein chains are colored by structure (*α*‐helix green, extended‐*β* yellow, turn orange, coil red).

The pictures in **Figure** **30** show the 18 positions modeled for departing NH_3_. At each NH_3_ position, the N (grey) and H (cyan) atoms and their hydrogen bonds are marked, but H atoms on the water molecules and their hydrogen bonds are not shown, for clarity. Detailed descriptions and explanations are contained in ref. [22]. Beyond the last modeled position, near Gln53A, there is a “river‐delta” of possibilities for NH_3_ to escape to the protein surface. This is evident in **Figure** **31** which summarizes the sequence of NH_3_ locations in relation to the associated waters and to the local protein, colored by structure (*α*‐helix green, extended‐*β* yellow, turn orange, and coil red), revealing the loose tertiary structure around most of the proposed NH_3_ pathway.

The species moving along the blue river was modeled as NH_3_ rather than NH_4_^+^ for two reasons. First, at positions 3 and 4, and then at positions 12, 13, 14, and 15, there are CH‐‐NH_3_ contacts involving the sidechains Ala65A, Gln191A, and Thr58A (Figure 30 and 31). These contacts, probably weak CH→NH_3_ hydrogen bonds, are too short (2.6—2.9 Å) to be the necessarily longer CH‐‐HNH_3_^+^ interactions. Secondly, the skipping movements involve repeated breaking and making of hydrogen bonds, and so the stronger hydrogen bonds formed by NH_4_^+^ could increase skipping barriers.

Rationalization of structure and function of the blue river is clear for the MoFe proteins. Analysis has not been made for the different blue river in the VFe protein. Finally, it is worth noting in the present context that there has been extensive research on the amidotransferase enzymes that transport ammonia through long channels, up to 40 Å, and that water is involved, structurally and functionally.^[^ ^43^, ^44^, ^45^, ^46^, ^47^, ^48^ ^–^ ^49^ ^]^

## 10 Northeast Water Pool

**Figure 32:**
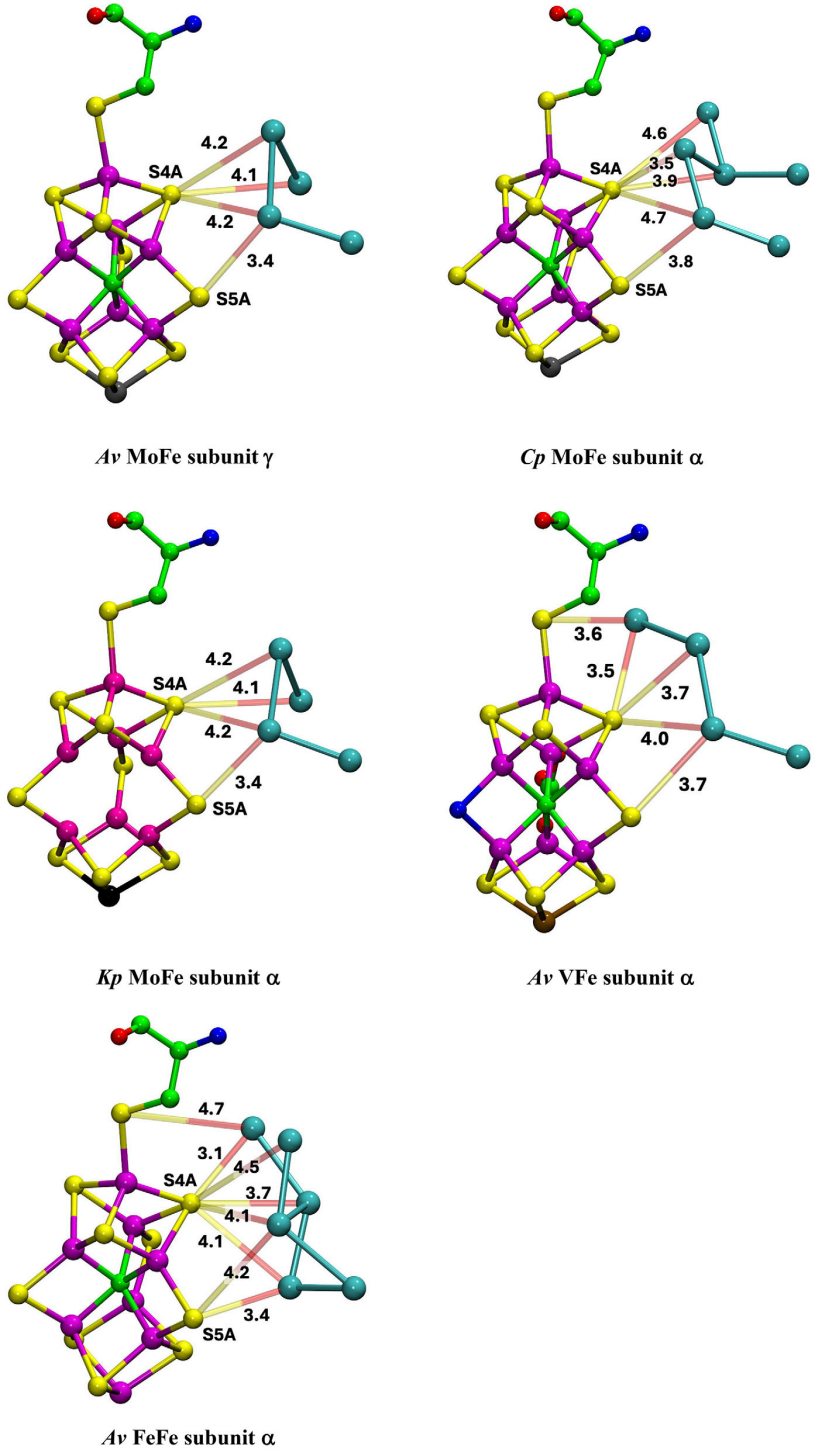
Five occurrences of the north east small water pool (cyan), from PDB entries 3U7Q, 4WES, 1QGU, 5N6Y, and 8BOQ. Distances < 3.2 Å within each pool are marked as bonds. Distances from water to atoms S5A, S4A, and Cys S are marked.

A collection of water molecules to the north east of the catalytic cofactor occurs in the crystal structures of all component 1 proteins of Mo‐, V‐, and Fe‐nitrogenases. This small water pool is variable in composition and position, adjacent to atoms S5A and S4A of the cofactor and far from homocitrate. **Figure** **32** shows these waters and their positions for the three species of MoFe protein, the *Av* VFe protein, and the *Av* FeFe protein. Note that (1) in the *Av* and *Kp* MoFe proteins, the pools of four waters are similar, but *Cp* contains two addition waters; (2) the VFe protein contains four waters but in a different position to those in the MoFe proteins; (3) the FeFe protein contains six waters; (4) distances and angles are consistent with intrapool hydrogen bonds in the MoFe and VFe proteins, but some of the contacts in the FeFe protein are angularly inappropriate for hydrogen bonds; (5) all pools have a water 3.4–3.8 Å from S5A of the cofactor, indicative of an O‐H → S hydrogen bond; (6) all pools contain three waters within hydrogen bonding distances of S4A, but the angular arrangements indicate limited hydrogen bonding to S4A as acceptor.

**Figure 33:**
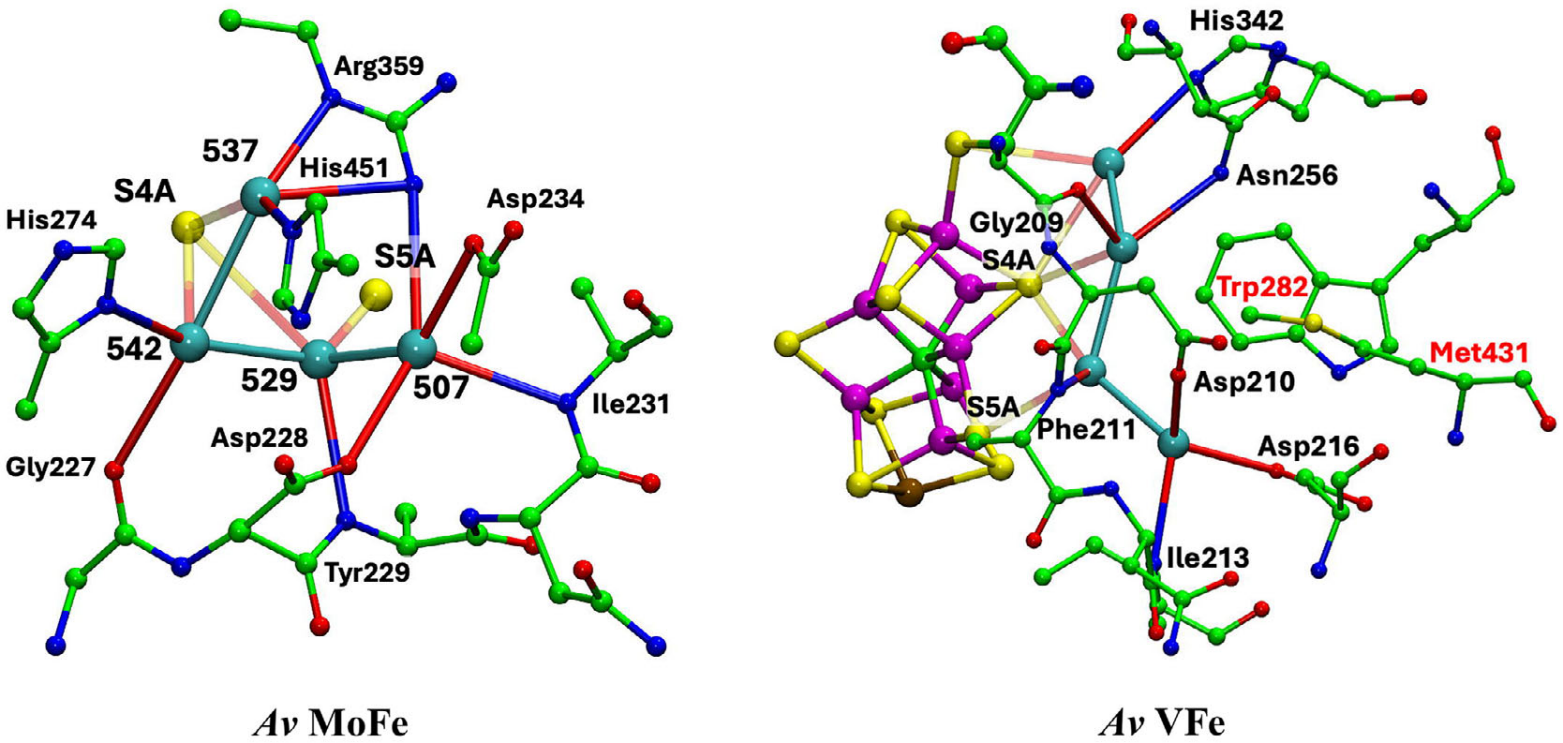
Hydrogen bonding possibilities for the northeast water pool with surrounding residues and S4A and S5A. In the FeV protein, the sidechains of Trp282 and Met431 form a hydrophobic wall to the water chain. The rear CO_3_ ligand of FeV‐co is deleted for clarity.

These pools occur in unstructured protein enclosures, with multiple hydrogen bonding distances between water and surrounding amino acids. **Figure** **33** shows two examples of this coordination for the *Av* MoFe protein and *Av* VFe protein. Irregular stereochemistry occurs for some water molecules: one water near S4A has near‐planar rather than pseudo‐tetrahedral contacts. On the eastern boundary of the water pools, there is usually a hydrophobic wall, illustrated by the Trp and Met sidechains labeled in red on the VFe protein in Figure 33.

**Figure 34:**
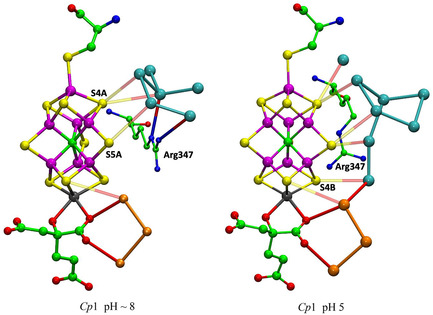
Structures of the MoFe *Cp*1 protein at pH ca 8 (PDB 4WES) and pH 5 (PDB 5VPW).

Morrison et al.^[^ ^50^ ^]^ reported the structure of *Cp* MoFe protein crystals soaked in pH 5 buffer and observed changes in the vicinity of the northeast water pool. Relevant parts of this structure, compared with the physiological structure (pH ≈8), are shown in **Figure** **34**. Even though the low pH structure is lower resolution (1.85 vs 1.08 Å) and has a smaller number of water molecules determined (527 vs 2484), additional waters are in the northeast pool, which extends south to within hydrogen bonding distance of W1 in the orange water chain. There is also an additional contact with S4B. Residue Arg347, which forms hydrogen bonds from its sidechain to two pool waters in the physiological crystal, flips to a non hydrogen bonding conformation in the acidified crystal (see Figure 34).

Previously, I reported an investigation of these MoFe proteins in which I patched in H atoms not observed in the X‐ray diffraction analyses and optimized various possible structures using density functional calculations on large models (463 atoms) that contained all surrounding residues: Arg96, the Ile226 to Ser238 chain, His274, Cys275, Asn298 to Phe300, Leu358, Arg359, His362, His442 to Trp444, Pro449 to Gly452. With validation of calculated structures against experimental geometry in the crystal structures, this yielded the most probable distributions of hydrogen atoms in the hydrogen bonds of the low‐ and high‐pH *Cp* MoFe structures and a location for an H_3_O^+^ in the low‐pH state.^[^ ^51^ ^]^

The collected results, previous and present, indicate that the northeast water pool probably has no mechanistic significance. This conclusion is based on (1) variability of composition and geometry of the northeast water pool in the various protein structures, (2) the occurrence of irregular geometry around some water molecules, and (3) the absence of linkage to a replenishable proton source in the physiological protein structures.

## 11 Single Water behind His

**Figure 35:**
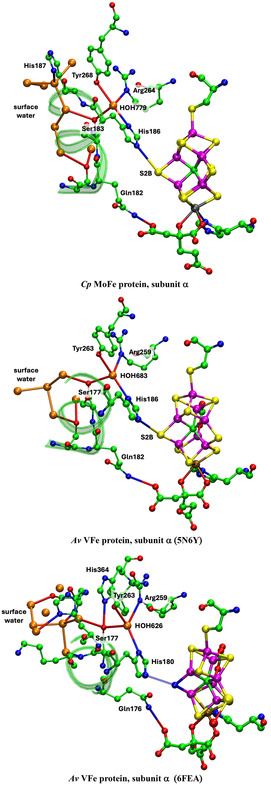
Environment of the single water molecule hydrogen bonded to N*δ* of the histidine residue that also forms a hydrogen bond to S2B via N*ε.* In the *Cp* MoFe protein (PDB 4WES), water 779 is hydrogen bonded well with good tetrahedral stereochemistry to His186 and the sidechains of Arg264, Tyr268, and Ser183. The hydrogen bonded network extends from OH of Ser183 into an extended set of surface waters (orange). The helix from Gln182 to His187 is marked. Corresponding representations of HOH683 in the *Av* VFe protein (PDB 5N6Y) and of HOH626 in the VFe protein, nonresting state (PDB 6FEA), are included.

The overall water distributions described in Section 4 include a water molecule hydrogen bonded to N*δ* of the His residue that also forms a hydrogen bond via N*ε* to S2B of the cofactor. This single water molecule is conserved in all protein structures, including the putative turnover state structures. **Figure** **35** shows the details for the *Cp* MoFe (4WES) and *Av* VFe (5N6Y) proteins and the putative turnover state of the *Av* VFe protein (6FEA).

This single water molecule (now dubbed “His‐water”) and its surrounding residues, comprising the histidine reside that connects to the cofactor, and the sidechains of arginine, tyrosine and serine, are strictly conserved in all proteins. The four hydrogen bonds involving His‐water are standard lengths, and the stereochemistry is close to tetrahedral. The OH sidechain of the serine residue has an additional hydrogen bond into the array of water molecules on the protein surface. Naturally, the surface water structures are variable and depend on the identity of the protein, but His‐water, its environs, and its connection to the cofactor via His and S2B are absolutely conserved.

**Figure 36:**
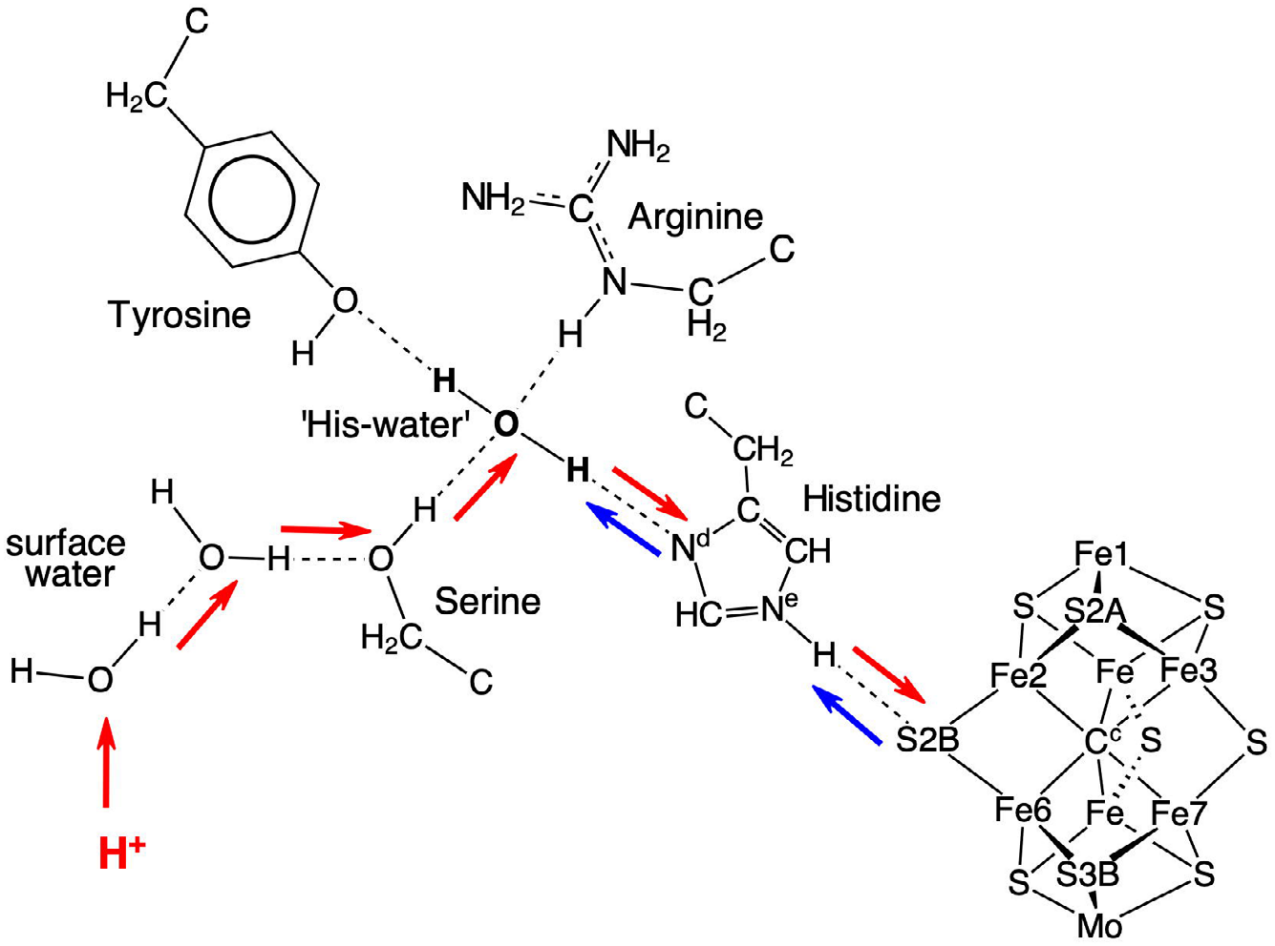
Conserved residues forming the environment of the single water molecule, “His‐water,” hydrogen bonded to the back side (N*δ*) of histidine. Possible proton slides link surface water to proton buffering at S2B.

I postulate that this structural feature enhances, and possibly controls, the proton buffering function of the histidine sidechain to S2B and the reaction zone of the cofactor. This functionality is illustrated in **Figure** **36**. An external proton can initiate a series of proton slides (red arrows) to protonate N*δ* of histidine, enhancing a proton slide from N*ε* of histidine to S2B. The reverse can occur, and a proton on S2B could move back to N*ε* and then from N*δ*‐H to His‐water (blue arrows). In this way, histidine is functioning as a proton buffer for S2B, facilitated by the reversible slide of protons along the chain via serine to the protein surface. Tyrosine and arginine are unchanged. This His‐water link between the cofactor and the protein surface traverses the north western desert, as is evident in Figures 2 and 3.

Note that this buffer mechanism (surface‐water ⇔ His‐water ⇔ HisH^+^ ⇔ S2B) does not supply surface protons to S2B and the cofactor. Such supply would require 180° rotation of the histidine side chain, which is energetically and sterically very difficult.^[^ ^52^ ^]^ In my proposed complete mechanism for the conversion of N_2_ to 2NH_3_ by Mo‐nitrogenase, there are two occurrences of the proton buffer role of His195 and protons moving to and from S2B.^[^ ^25^ ^,^ ^26^ ^,^ ^28^ ^]^

## 12 Comparison with Cytochrome c Oxidase

**Figure 37:**
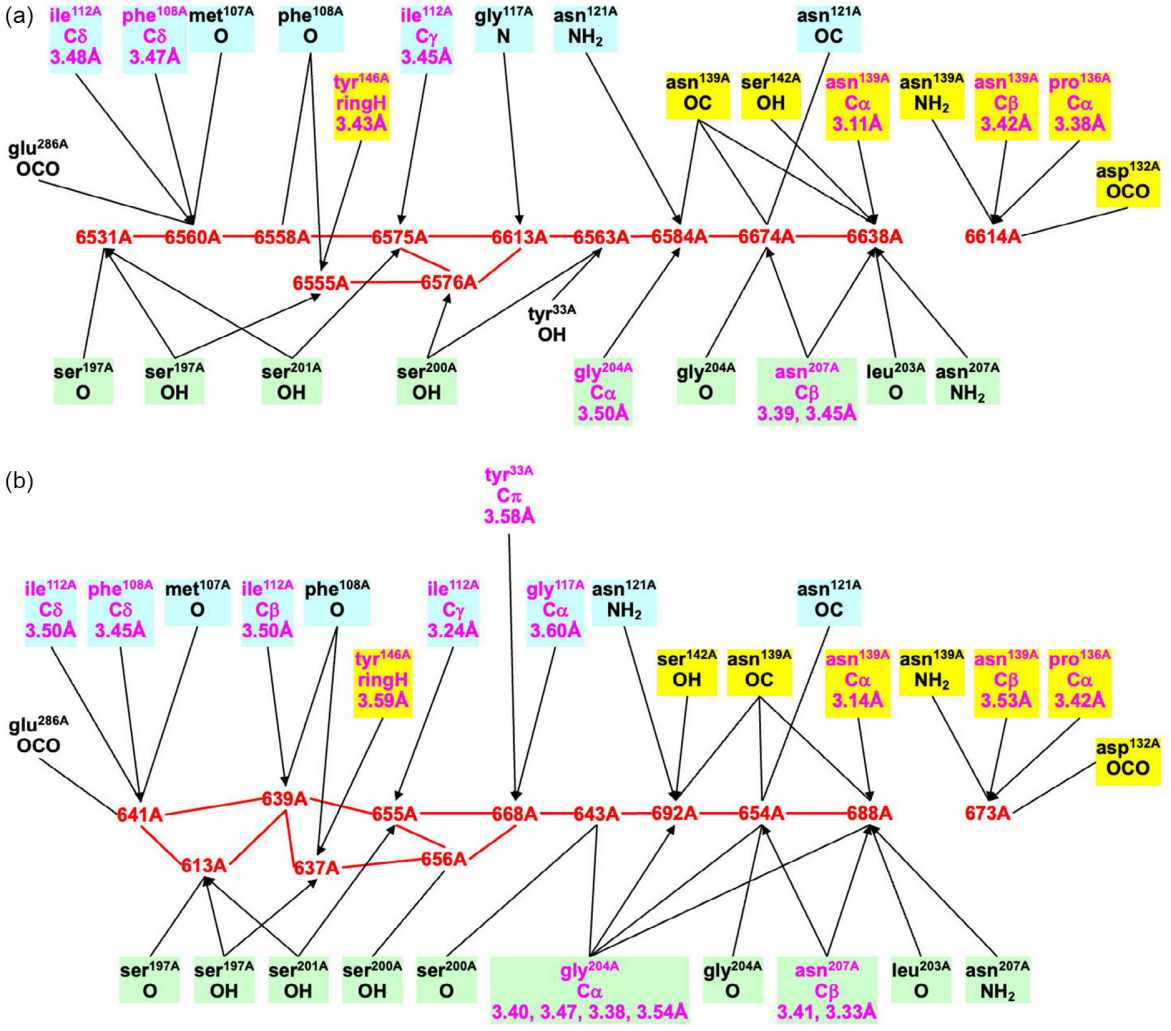
The “D‐pathway” for proton pumping in two crystal structures of cytochrome c oxidase from *Rhodobacter sphaeroides.* a) PDB 2GSM, 2.0 Å resolution. b) PDB 3FYE, 2.15 Å resolution. In both cases, the proton pump direction is right to left, towards 286Glu. 132^Asp^ marks the entrance to the pathway, but the water wire commences at the second water molecule. As in the previous maps, residues from the same section of protein chain are colored similarly, and weaker longer C–H••O and O••C*π* interactions are colored magenta.

The elaborately functional water features of the nitrogenase proteins can be compared with another complex system with dual functionality, the membrane metalloenzyme cytochrome c oxidase (CcO). Nitrogenase uses separate water paths to introduce protons required to reduce the substrate and to remove the product of reduction, while CcO translocates protons to reduce substrate O_2_ to water (no removal of product being required) and also to pump protons across the membrane to maintain a trans‐membrane proton gradient.^[^ ^16^ ^,^ ^18^ ^,^ ^53^, ^54^ ^–^ ^55^ ^]^ It is instructive to compare the proton translocation structural chemistry in these two systems. **Figure** **37** maps the water chains and surrounds in two crystals of cytochrome c oxidase from the bacterium *Rhodobacter sphaeroides*, in an oxidized form (PDB 2GSM, 2.0 Å resolution)^[^ ^56^ ^]^ and reduced form (PDB 3FYE, 2.15 Å resolution):^[^ ^57^ ^]^ the pumping direction is right to left. The main message to take from this is that the proton pumping system of cytochrome c oxidase and the proposed proton pumping system of nitrogenase are structurally similar. In both cases, there is a sequence of hydrogen bonded water molecules, with some branching, enclosed with strongly hydrogen bonding functions from the main chain (O) and side chains (OH from tyrosine, serine, NH_2_ from arginine, asparagine, carboxylate from aspartate). A more distant sheath of weakly hydrogen bonding C–H and C*π* functions is present in both cases. The main difference between the proton pathways in nitrogenase and cytochrome c oxidase is the more frequent occurrence of main‐chain N‐H•••water hydrogen bonds in the nitrogenase pathways. Nevertheless, it is clear that very similar structural features are deployed in nitrogenase and cytochrome c oxidase to effect vectorial translocation of protons. Note that the hydrogen bonds of H_3_O^+^ can be accommodated at the water positions along chains of cytochrome c oxidase and that detailed proton transfer steps along the chain, as proposed for nitrogenase up to W3, can be constructed for cytochrome c oxidase.

## 13 Dynamic Proteins

The protein water structures described above are derived from static crystal structures. But, since protein structures are dynamic, and fluctuations in protein tertiary structure can be intimately connected with enzyme mechanism, a key question arises. How do the water features of the nitrogenase component 1 proteins change during the mechanistic cycle? The form of V nitrogenase reported in structure 6FEA is postulated to be a “turnover state” by the authors, but, because the crystals took about one week to grow,^[^ ^32^ ^]^ the catalytic relevance of the crystal structure is uncertain. The crystal structure 8BOQ of Fe nitrogenase is also proposed to be a crystallographically isomorphous mixture of resting and turnover state, but again it took 4 days for crystals to appear.^[^ ^33^ ^]^

Crystal growth, inevitably much slower than turnover, could be used with confidence if an intermediate could be trapped and stored under crystallization conditions. Intermediates in the nitrogenase catalytic cycle notoriously defy isolation, for several reasons, one of which is that protons are a substrate, being reduced to H_2_. Fortunately, the technique of cryo‐electron microscopy (cryoEM) can capture and analyze samples on the turnover time scale. Warmack and Rees report cryoEM analysis of Mo‐nitrogenase under alkaline (pH 9.5) conditions with acetylene as substrate.^[^ ^58^ ^]^ This system was designed to slow the rate and to suppress proton reduction. CryoEM structures were reported for reaction times 20 s (PDB 9CJE), 5 min (PDB 9CJD), 20 min (PDB 9CJC), and 60 min (at which only 17% activity remained). They estimate the 20 s time point could capture the initial turnover event. The resulting protein structures were resolved to ca 2 Å.

These results allow an approach to the question raised above about the water features of the protein during the mechanistic cycle. With reference to the resting state MoFe protein and focusing on the cryoEM structures at 20 s (PDB 9CJE), 5 min (PDB 9CJD), and 20 min (PDB 9CJC), I make the following observations. (1) Waters W1 to W8 of the proton wire are present at 20 s and at 5 min, but at 20 min, W4 is missing. (2) The outer section of the orange water path shows variation in all structures. (3) The homocitrate pool (yellow) varies in compositions and arrangement. (4) The northeast water pool contains 3 waters at 20 s and 4 waters at 5 min and 20 min. (5) The single water behind His 195 (His‐water) is retained throughout. (6) The water molecule hydrogen bonding between Nε of His442 and side chain carboxylate of Asp380 is retained.

The waters in the blue river manifest variation and disruption at all time points. This blue river disruption correlates with the observations of Warmack and Rees that at all time points there is disordering in the *α*III domain (residues *α*25 to *α*48 and *α*378 to *α*403), because the blue river is adjacent to the *α*III domain. The Warmack‐Rees experiments generated ethylene as enzyme product, a molecule that is hydrophobic and larger than physiological product ammonia. A suggested interpretation is that the *α*III domain moves aside to create space for the departing ethylene and that the blue river is less structured because it is not required to host a departing hydrophylic product.

Rutledge et al.^[^ ^59^ ^]^ obtained cryoEM data for the MoFe protein with the Fe protein docked, on active samples prepared within 30 s of initiation of turnover (PDB 7UT8, 7UT9). Nominal resolutions were 2.4 Å, but only a few water molecules were resolved or reported and none are near FeMo‐co or the other water structures of the resting state protein.

## 14 Summary and Conclusion

This survey of the presence and structures of water molecules within the nitrogenase proteins draws attention to features not previously recognized, and elaborates the prevalence of features already described. The exploration centers on the catalytic cofactor and extends to the hydration shell covering the component 1 proteins that contain the cofactor. Information is sourced from crystal structures reported in the protein database. The three isozymes, Mo‐, V‐, and Fe‐nitrogenase, from the species *Av*, *Cp*, and *Kp*, are included where data is available. Some features occur in all reported structures, and some are species invariant but isozyme dependent, as summarised in **Table** **2**. Interpretations of the intraprotein water constituents and the considerations that arise about their functionality in the chemical mechanisms of the enzyme are discussed. The structure of water on protein surfaces is well studied^[^ ^60^ ^,^ ^61^ ^]^and not discussed here, other than to note the general result that the sites where the Fe protein (component 2) docks with the component 1 protein are not near any of the surface positions described here, being approximately on the side of the P‐cluster opposite to the cofactor.^[^ ^62^ ^]^

**Table 2:** Summary of water features and occurrences.

Water feature	MoFe proteins	VFe protein
Inner proton wire water pathway, W1 to W8	Strictly conserved in *Av*, *Cp*, *Kp* proteins	W1, W2, and W3 only in *Av* proteins
Outer water path and proton bay	Present in *Av*, *Cp*, *Kp* proteins, variable composition and position	Very different from MoFe proteins
“His water”	Present in *Av*, *Cp*, *Kp* proteins, conserved connections to 4 surrounding residues	Present in *Av* proteins, conserved connections to 4 surrounding residues
Ammonia river	Present in *Av*, *Cp*, *Kp* proteins with small species variations	Present in *Av* protein, partly similar to MoFe protein
Water hydrogen bonded to N*ε*H of ligand histidine	Single water in *Av*, *Cp*, *Kp* proteins	Chain of eight waters
Homocitrate water pool	Present in *Av*, *Cp*, *Kp* proteins with large species variations in composition and structure	Present in *Av* protein, less extensive than MoFe protein
Northeast water pool	Present in *Av*, *Cp*, *Kp* proteins with species variations in composition and structure	Present in *Av* protein, different from MoFe protein

1) The water features of the nitrogenase proteins are structurally different and more varied in function than those of other enzymes and biochemical systems. Here I summarize the structural and functional properties supporting this assertion.

2) Deserts—large volume anhydrous regions within the proteins—occur in all nitrogenase crystal structures with resolution sufficient to conclude that few if any water molecules have been missed in the crystallographic analyses.

3) The deserts occur in one protein subunit and cover large sectors around the cofactor: deserts are west, northwest, behind, and east of the cofactor.

4) The few (3–5) buried waters in the deserts are distant (ca 10 Å) from the cofactor and are too distant to function in the catalytic mechanism. Statistically, the number of buried waters in the deserts is approximately normal.

5) The reaction zone for the catalytic mechanism of nitrogenase is totally devoid of water. It is not plausible to invoke water in the reaction space. Postulation of unidentified water as a protonation agent near bound N_2_ or other intermediates in not valid.

6) Proton terminology refers to an H atom, polarized slightly positive by its bond to a more electronegative atom. Protons and H^‐^ ions (2 electrons) cannot exist as such in condensed media, including protein and water, or on atoms of the metal sulfide cluster at the catalytic site.^[^ ^29^ ^]^

7) All proteins contain “His‐water,” a single water molecule hydrogen bonded to the backside N*δ* of an absolutely conserved histidine residue that can also hydrogen bond (via N*ε*) with S2B in the reaction zone.

8) The four hydrogen bonding partners for His‐water are totally conserved in all proteins.

9) His‐water is postulated to have a mechanistically significant role, involving protons shuttling from the aqueous protein surface to N*δ* of the histidine sidechain, influencing its charge status, and thereby influencing proton transfer between N*ε* and S2B. This is occurs twice in the proposed mechanistic cycl.^25^, ^26^, ^28^

10) The His‐water mechanism functions to buffer proton transfer with S2B but cannot supply the replenishable protons required for the nitrogenase mechanism cycle.

11) The VFe and FeFe proteins have a single water molecule hydrogen bonded between S5A and the sidechain of a Lys residue (83 in VFe). In the MoFe proteins, this residue is Arg96 which hydrogen bonds directly with S5A from its guanidine sidechain, and there is no intervening water. The sidechain of this Arg or Lys residue flanks the reaction zone and in all proteins can swing aside to expand this reaction space without disrupting the hydrogen bonding with S5A.^[^ ^28^ ^]^

12) The small water pool to the north east of the cofactor in MoFe and VFe proteins contains 4–6 water molecules, hydrogen bonded to each other and possibly with atoms S4A and/or S5A of the cofactor.

13) The composition and structure of the small water pool are variable. There is no evident mechanistic function for this water aggregate. There is no source of replenishable protons. This northeast pool is classified as a billabong, containing stagnant space‐filling water.

14) All nitrogenase component 1 proteins contain two elongated sequences of hydrogen bonded water molecules, classified as rivers.

15) The two rivers extend in opposite directions from the homocitrate end of the cofactor.

16) One river is interpreted as the water path along which the protons required by the enzyme are transported to the cofactor from the aqueous environment of the protein surface. This is the proton transporting river, able to provide sequentially the multiple protons (8 under physiological conditions) required in each catalytic cycle of the enzyme.

17) In the MoFe proteins, the proton transporting water chain has two sections. The inner section near the cofactor contains eight absolutely conserved water molecules, while continuation of the water chain towards the protein surface is variable. The inner section, W1 to W8, is the strictly controlled proton wire, and outer section is regarded as a possible holding bay for protons, as H_2n + 1_O_n_^+^ species. Small exogenous hydrophilic molecules are included in two of the crystals of MoFe protein, signifying accessibility from the protein surface.

18) Three carboxylate O atoms of homocitrate, O6, O5, and O3, are essential hydrogen bonding anchors for waters W1, W3, and W4 of the proton water chain. A conserved glycine carbonyl O provides two hydrogen bond acceptor sites and a conserved arginine side chain two donor sites, to anchor waters W3, W4, W5, and W6.

19) There is a standard Grotthuss mechanism for translocation of protons from W8 to W1 along the proton wire and finally to S3B of the cofactor. This mechanism has been been described in full detail using density functional calculations. The alternating HOH rotations and H atom slides have low potential energy barriers, <7 kcal mol^−1^.

20) Electron transfer to the cofactor is proposed as the trigger for the last stage of proton transfer from W1 to S3B, reducing the kinetic barrier by ca 3.5 kcal mol^−^^1^ and stabilizing the product by ca 7 kcal mol^−^^1^.

21) In the VFe protein, the water structure in the proton transfer river is markedly different from that of the MoFe proteins. The closest waters W1, W2, and W3 are the same in both, but in the VFe protein, there is not a continuous sequence of water molecules to the protein surface. An alternative Grotthuss proton translocation mechanism, possibly less efficient, is proposed for the vanadium proteins.

22) A second river emanates from the cofactor in a direction opposite to the proton path river. It has the function of aiding egress of product NH_3_ and is referred to as the ammonia river. This opposite directionality of the ammonia river and the proton river provides separation of the basic and acidic components of the nitrogenase reaction. Traffic on the proton river is from protein surface towards the cofactor, while traffic on the ammonia river is from cofactor to surface.

23) The ammonia rivers are not continuous hydrogen bonded sequences of water molecules. Migrating NH_3_ molecules do not replace water molecules in this river but swing through a sequence of positions by relinquishing previous hydrogen bonds and forming new hydrogen bonds with river water and surrounding residues.

24) Calculations of cavities in the MoFe protein revealed the continuous sequence of pockets that define the ammonia river.

A mechanism has been described for the passage of NH_3_ after its dissociation from cofactor to near the protein surface. This mechanism for the MoFe proteins is comprised of 18 hydrogen bonded intermediates. The first stages of NH_3_ movement away from the cofactor are into a hydrogen bonding environment comprised of homocitrate carboxylate atoms O1, O2, and O4 and water molecules.

25) In the MoFe proteins, the three carboxylate groups of homocitrate have connections with the proton river and the ammonia river and also separate them. Carboxylate O5, O6, and O3 support the proton river, and O1, O2, and O4 support the ammonia river.

26) In the VFe protein, the beginning of the ammonia river is markedly different from that in the MoFe proteins. In VFe, there is a sequence of 8 waters, connected by a hydrogen bond from N*ε*H of the histidine (423 in *Av*1) that is also ligated to V in the cofactor. In all MoFe proteins, there is only one water hydrogen bonded to the corresponding histidine.

27) There are similarities and differences between the V and Mo ammonia rivers as they wind towards the protein surface. A mechanism for NH_3_ departure through the variant ammonia river in the VFe protein has not yet been detailed.

28) There is a third collection of water molecules (here dubbed “yellow water”) around homocitrate and forming some hydrogen bonds with it. Near homocitrate, there are some hydrogen bonding distances between yellow water molecules and water at the beginning of the ammonia river, but there are no hydrogen bonds between yellow water and the conserved section of the proton river.

29) The yellow water pool in the MoFe proteins contains 20—24 molecules, larger than in the VFe protein (ca 12), and extends into a region of unstructured protein. The yellow water aggregate in the VFe protein is blocked by an additional helical protein chain. The yellow water pools do not reach the protein surface and are buried.

30) The arrangements of water molecules in the yellow pools are all different. There are many water–water contacts < 3.2 Å, but some are angularly unsuited to hydrogen bonding. Some crystallographic disorder is reported. It appears that some fluidity could be present. The yellow water pool is suggested to function only as a water reservoir.

31) Protein structure around each of the water features in the nitrogenase proteins is presented pictorially.

32) Recent cryoEM data provides some insight into water structures during turnover of the Mo enzyme with acetylene. The inner section of the proton wire shows minor changes, and “His‐water” is unchanged, consistent with the proposed functionality. The ammonia river is disrupted, and the adjacent *α*III protein domain becomes disordered: both changes are consistent with the provision of space for the egress of ethylene, a larger and hydrophobic product.

33) Water metaphors—desert, river, pool, billabong, waterfall, gorge, and oases—have value in appreciating and understanding the water chemistry of nitrogenase component 1 proteins.^[^ ^63^ ^]^

## Conflict of Interest

The author declares no conflict of interest.

## References

[1] P. S. Nutman , Phil. Trans. Royal Soc. London B 1987, 317, 69.

[2] Y. Hu , M. W. Ribbe , Methods Mol. Biol. 2011, 766, 3. 21833857 10.1007/978-1-61779-194-9_1

[3] D. C. Rees , Ann. Rev. Biochem. 2002, 71, 221. 12045096 10.1146/annurev.biochem.71.110601.135406

[4] D. C. Rees , F. A. Tezcan , C. A. Haynes , M. Y. Walton , S. Andrade , O. Einsle , J. A. Howard , Phil. Trans. Royal Soc. A 2005, 363, 971. 10.1098/rsta.2004.1539 15901546

[5] T. Spatzal , M. Aksoyoglu , L. Zhang , S. L. A. Andrade , E. Schleicher , S. Weber , D. C. Rees , O. Einsle , Science 2011, 334, 940. 22096190 10.1126/science.1214025 PMC3268367

[6] K. M. Lancaster , M. Roemelt , P. Ettenhuber , Y. Hu , M. W. Ribbe , F. Neese , U. Bergmann , S. DeBeer , Science 2011, 334, 974. 22096198 10.1126/science.1206445 PMC3800678

[7] T. Spatzal , Z. Anorg. Allg. Chem. 2015, 641, 10.

[8] O. Einsle , D. C. Rees , Chem. Rev. 2020, 120, 4969. 32538623 10.1021/acs.chemrev.0c00067 PMC8606229

[9] I. Dance , Chembiochem 2020, 21, 1671. 31803989 10.1002/cbic.201900636

[10] S. D. Threatt , D. C. Rees , FEBS Lett. 2022, 597, 45. 36344435 10.1002/1873-3468.14534 PMC10100503

[11] R. A. Warmack , D. C. Rees , Molecules 2023, 28, 7952. 38138444 10.3390/molecules28247952 PMC10745740

[12] R. R. Eady , Coord. Chem. Rev. 2003, 237, 23.

[13] C. A. Wraight , Biochim. Biophys. Acta—Bioenerg. 2006, 1757, 886. 10.1016/j.bbabio.2006.06.017 16934216

[14] P. Ball , Chem. Rev. 2008, 108, 74. 18095715 10.1021/cr068037a

[15] C. Knight , G. A. Voth , Acc. Chem. Res. 2012, 45, 101. 21859071 10.1021/ar200140h

[16] H. J. Lee , J. Reimann , Y. Huang , P. Ädelroth , Biochim. Biophys. Acta—Bioenerg. 2012, 1817, 537. 10.1016/j.bbabio.2011.10.007 22056517

[17] Y. Wuxiuer , E. Morgunova , N. Cols , A. Popov , A. Karshikoff , I. Sylte , R. Gonzàlez‐Duarte , R. Ladenstein , J.‐O. Winberg , FEBS J. 2012, 279, 2940. 22741949 10.1111/j.1742-4658.2012.08675.x

[18] S. Yoshikawa , K. Muramoto , K. Shinzawa‐Itoh , Ann. Rev. Biophys. 2011, 40, 205. 21545285 10.1146/annurev-biophys-042910-155341

[19] B. K. Mai , K. Park , M. P. T. Duong , Y. Kim , J. Phys. Chem. B 2013, 117, 307. 23234421 10.1021/jp310724g

[20] Y. Matsuki , M. Iwamoto , K. Mita , K. Shigemi , S. Matsunaga , S. Oiki , J. Am. Chem. Soc. 2016, 138, 4168. 26959718 10.1021/jacs.5b13377

[21] I. Dance , Dalton Trans. 2012, 41, 7647. 22609731 10.1039/c2dt30518f

[22] I. Dance , Sci. Rep. 2013, 3, 3237. 24241241 10.1038/srep03237 PMC3831235

[23] I. Dance , Dalton Trans. 2015, 44, 18167. 26419970 10.1039/c5dt03223g

[24] R. R. Eady , Chem. Rev. 1996, 96, 3013. 11848850 10.1021/cr950057h

[25] I. Dance , Dalton Trans. 2024, 53, 14193. 39140218 10.1039/d4dt01866d

[26] I. Dance , Dalton Trans. 2024, 53, 19360. 39513199 10.1039/d4dt02606c

[27] I. Dance , Dalton Trans. 2025, 54, 3013. 39812693 10.1039/d4dt03146f

[28] I. Dance , Dalton Trans. 2025, 54, 9877. 40470848 10.1039/d5dt00658a

[29] I. Dance , Dalton Trans. 2015, 44, 9027. 25891439 10.1039/c5dt00771b

[30] S. M. Mayer , D. M. Lawson , C. A. Gormal , S. M. Roe , B. E. Smith , J. Mol. Biol. 1999, 292, 871. 10525412 10.1006/jmbi.1999.3107

[31] D. Sippel , O. Einsle , Nat. Chem. Biol. 2017, 13, 956. 28692069 10.1038/nchembio.2428 PMC5563456

[32] D. Sippel , M. Rohde , J. Netzer , C. Trncik , J. Gies , K. Grunau , I. Djurdjevic , L. Decamps , S. L. A. Andrade , O. Einsle , Science 2018, 359, 1484. 29599235 10.1126/science.aar2765

[33] C. Trncik , F. Detemple , O. Einsle , Nat. Catal. 2023, 6, 415.

[34] O. Carugo , Curr. Protein Peptide Sci. 2015, 16, 259. 25723549 10.2174/1389203716666150227162803

[35] O. Carugo , Amino Acids 2016, 48, 193. 26315961 10.1007/s00726-015-2064-4

[36] C. J. T. de Grotthuss , Ann. Chim. 1806, 58, 54.

[37] N. Agmon , Chem. Phys. Lett. 1995, 244, 456.

[38] R. Pomès , B. Roux , Biophys. J. 1998, 75, 33. 9649365 10.1016/S0006-3495(98)77492-2 PMC1299677

[39] A. Migliore , N. F. Polizzi , M. J. Therien , D. N. Beratan , Chem. Rev. 2014, 114, 3381. 24684625 10.1021/cr4006654 PMC4317057

[40] I. Dance , Dalton Trans. 2024, 53, 7996. 38651170 10.1039/d4dt00474d

[41] R. M. Stroud , L. J. W. Miercke , J. O’Connell , S. Khademi , J. K. Lee , J. Remis , W. Harries , Y. Robles , D. Akhavan , Curr. Opin. Struct. Biol. 2003, 13, 424. 12948772 10.1016/s0959-440x(03)00114-3

[42] V. L. Guilloux , P. Schmidtke , P. Tuffery , BMC Bioinf. 2009, 10, 168. 10.1186/1471-2105-10-168 PMC2700099 19486540

[43] R. H. van den Heuvel , B. Curti , M. A. Vanoni , A. Mattevi , Cell. Mol. Life Sci. 2004, 61, 669. 15052410 10.1007/s00018-003-3316-0 PMC11138638

[44] A. Weeks , L. Lund , F. M. Raushel , Curr. Opin. Chem. Biol. 2006, 10, 465. 16931112 10.1016/j.cbpa.2006.08.008

[45] M. A. Vanoni , B. Curti , IUBMB Life 2008, 60, 287. 18421771 10.1002/iub.52

[46] A. Nakamura , M. Yao , S. Chimnaronk , N. Sakai , I. Tanaka , Science 2006, 312, 1954. 16809541 10.1126/science.1127156

[47] J. Wu , W. Bu , K. Sheppard , M. Kitabatake , S.‐T. Kwon , D. Söll , J. L. Smith , J. Mol. Biol. 2009, 391, 703. 19520089 10.1016/j.jmb.2009.06.014 PMC2830067

[48] S. Mouilleron , B. Golinelli‐Pimpaneau , Curr. Opin. Struct. Biol. 2007, 17, 653. 17951049 10.1016/j.sbi.2007.09.003

[49] J. Kang , S. Kuroyanagi , T. Akisada , Y. Hagiwara , M. Tateno , J. Chem. Theory Comput. 2012, 8, 649. 26596613 10.1021/ct200387u

[50] C. N. Morrison , T. Spatzal , D. C. Rees , J. Am. Chem. Soc. 2017, 139, 10856. 28692802 10.1021/jacs.7b05695 PMC5553094

[51] I. Dance , FEBS J. 2018, 285, 2972. 29797782 10.1111/febs.14519

[52] I. Dance , J. Inorg. Biochem. 2017, 169, 32. 28104568 10.1016/j.jinorgbio.2017.01.005

[53] K. Shimokata , Y. Katayama , H. Murayama , M. Suematsu , T. Tsukihara , K. Muramoto , H. Aoyama , S. Yoshikawa , H. Shimada , Proc. Nat. Acad. Sci. 2007, 104, 4200. 17360500 10.1073/pnas.0611627104 PMC1820732

[54] H. J. Lee , E. Svahn , J. M. J. Swanson , H. Lepp , G. A. Voth , P. Brzezinski , R. B. Gennis , J. Am. Chem. Soc. 2010, 132, 16225. 20964330 10.1021/ja107244g PMC3005615

[55] H.‐Y. Chang , S. K. Choi , A. S. Vakkasoglu , Y. Chen , J. Hemp , J. A. Fee , R. B. Gennis , Proc. Nat. Acad. Sci. 2012, 109, 5259. 22431640 10.1073/pnas.1107345109 PMC3325665

[56] L. Qin , C. Hiser , A. Mulichak , R. M. Garavito , S. Ferguson‐Miller , Proc. Nat. Acad. Sci. 2006, 103, 16117. 17050688 10.1073/pnas.0606149103 PMC1616942

[57] L. Qin , J. Liu , D. A. Mills , D. A. Proshlyakov , C. Hiser , S. Ferguson‐Miller , Biochemistry 2009, 48, 5121. 19397279 10.1021/bi9001387 PMC2720787

[58] R. A. Warmack , D. C. Rees , Nat. Commun. 2024, 15, 10472. 39622820 10.1038/s41467-024-54713-0 PMC11612016

[59] H. L. Rutledge , B. D. Cook , H. P. M. Nguyen , M. A. Herzik , F. A. Tezcan , Science 2022, 377, 865. 35901182 10.1126/science.abq7641 PMC9949965

[60] M. Nakasako , Cell. Mol. Biol. 2001, 47, 767. 11728092

[61] M.‐C. Bellissent‐Funel , A. Hassanali , M. Havenith , R. Henchman , P. Pohl , F. Sterpone , D. van der Spoel , Y. Xu , A. E. Garcia , Chem. Rev. 2016, 116, 7673. 27186992 10.1021/acs.chemrev.5b00664 PMC7116073

[62] F. A. Tezcan , J. T. Kaiser , D. Mustafi , M. Y. Walton , J. B. Howard , D. C. Rees , Science 2005, 309, 1377. 16123301 10.1126/science.1115653

[63] W. Humphrey , A. Dalkie , K. Schulten , J. Mol. Graph. 1996, 14, 33. 8744570 10.1016/0263-7855(96)00018-5

[64] L.‐M. Zhang , C. N. Morrison , J. T. Kaiser , D. C. Rees , Acta Crystallogr. Sect. D 2015, 71, 274. 25664737 10.1107/S1399004714025243 PMC4321486

[65] M. Rohde , K. Grunau , O. Einsle , Angew. Chem. Int. Ed. 2020, 59, 23626. 10.1002/anie.202010790 PMC7756900 32915491

